# Mitochondrial Protein MjEF‐Tu is Secreted into Host Plants by Nematodes Eliciting Immune Signaling and Resistance

**DOI:** 10.1002/advs.202412968

**Published:** 2025-01-30

**Authors:** Borong Lin, Shaozhen Huang, Zhiwen Li, Qiuling Huang, Handa Song, Tianyi Fang, Jinling Liao, Godelieve Gheysen, Kan Zhuo

**Affiliations:** ^1^ College of Plant Protection South China Agricultural University Guangzhou 510642 China; ^2^ Guangdong Province Key Laboratory of Microbial Signals and Disease Control South China Agricultural University Guangzhou 510642 China; ^3^ Department of Biotechnology Ghent University Ghent 9000 Belgium

**Keywords:** Mitochondrial protein, Nematode, PAMPs, PAMP‐triggered immunity

## Abstract

Little is known about plant‐parasitic animal‐derived pathogen‐associated molecular pattern (PAMP)/ pattern‐recognition receptor (PRR) pairs. Additionally, mitochondrial proteins have not previously been reported to be secreted into hosts by pathogens. Here, it is found that the *Meloidogyne javanica* elongation factor thermo unstable (EF‐Tu) (MjEF‐Tu) located in the nematode mitochondria is up‐regulated and secreted into the host plant during nematode parasitism. MjEF‐Tu interacts with the PRR *Arabidopsis thaliana* EF‐Tu receptor (AtEFR), triggering the plant hallmark defence responses mediated by AtEFR. An 18‐aa sequence (Nelf18) in the N terminus of the nematode EF‐Tu contributes to the immunogenic activity. *M. javanica* water extract and mitochondrial extract also induce plant immunity sensed by AtEFR, owing to the presence of MjEF‐Tu. In addition, Nelf18 enhances plant resistance to nematode, virus, and bacterial infections depending on AtEFR. These findings first demonstrate that mitochondrial proteins from pathogens can be secreted into hosts and function as a cross‐kingdom signal and identified the first plant‐parasitic animal‐derived proteinaceous PAMP/PRR pair, providing novel insights into host‐pathogen interactions.

## Graphical abstract

This study reports that the mitochondrial protein EF‐Tu from *Meloidogyne javanica* is secreted into the host plant during nematode parasitism, where it interacts with the PRR AtEFR, thereby activating AtEFR‐mediated plant immune responses. This work represents the first demonstration that mitochondrial proteins from pathogens can act as cross‐kingdom signaling molecules and identifies the first plant‐parasitic animal‐derived proteinaceous PAMP/PRR pair.

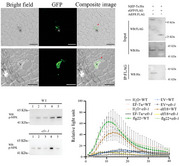

## 1 Introduction

Plants are exposed to various attackers, including pathogens, and have developed a two‐branched innate immune system to fight off pathogens.^[^ ^1^, ^2^ ^]^ The first branch is the pathogen‐associated molecular pattern (PAMP)‐triggered immunity (PTI) and the second branch is the effector‐triggered immunity (ETI). PTI is induced on the recognition of PAMPs by plant cell surface‐localised pattern‐recognition receptors (PRRs),^[^ ^3^ ^]^ activating an array of downstream immune responses. Generally, these responses include a burst of reactive oxygen species (ROS), activation of mitogen‐associated protein kinases (MAPKs), up‐regulation of defence‐related genes, and accumulation of callose.^[^ ^2^ ^]^

PAMPs are evolutionarily conserved molecules that exist in one or several classes of pathogens and play crucial roles in pathogen survival.^[^ ^4^, ^5^ ^]^ When pathogenic microorganisms invade host cells, PAMPs are perceived as nonself signals via PRRs, inducing the PTI responses. Well‐known PAMPs recognized by plants are bacterial flagellin, elongation factor thermo unstable (EF‐Tu), lipopolysaccharides and peptidoglycans; viral or bacterial nucleic acids; fungal cell wall‐derived chitin, glucans and mannans.^[^ ^4^, ^6^, ^7^ ^]^ Among these, the bacterial EF‐Tu has typical characteristics of PAMPs: high abundance, high sequence conservation, and essential for microbial survival.^[^ ^8^ ^]^ PAMP activity is attributed to a highly conserved 18‐amino acid (aa) sequence in the N‐terminus known as elf18.^[^ ^7^ ^]^ The bacterial EF‐Tu and elf18 are detected by the EF‐Tu Receptor (EFR), a PRR in Brassicaceae plants. EFR belongs to the receptor‐like kinase family and has an extracellular domain with 24 leucine‐rich repeats, one single transmembrane domain, and one intracellular serine/threonine kinase domain.^[^ ^3^ ^]^

Nematodes are the most abundant multicellular animals on Earth,^[^ ^9^ ^]^ with some of them parasitizing animals or plants. Plant‐parasitic nematodes (PPNs) annually cause losses of more than 70 billion dollars in crop yields.^[^ ^10^ ^]^ Among these, root‐knot nematodes (RKNs), *Meloidogyne* spp., are the most economically devastating PPNs.^[^ ^10^ ^]^ However, far less is known about PAMP/PRR pairs of plant‐parasitic animals. So far, only one non‐proteinaceous PAMP ascr#18 and its cognate PRR Arabidopsis NILR1 were found.^[^ ^11^ ^]^ Studies have reported that PTI responses in *Arabidopsis* are activated after treatment with nematode water extract (NemaWater).^[^ ^12^ ^]^ It was believed that PAMPs with a proteinaceous nature present in NemaWater, but no proteinaceous PAMPs have been identified yet.

An organism's EF‐Tu is involved in the GTP‐dependent binding of aminoacyl‐tRNA to the A site of ribosomes during protein synthesis, playing a vital role in survival. Different from bacterial EF‐Tu, EF‐Tu proteins of eukaryotic organisms are considered to reside in the mitochondria, delivering aminoacyl‐tRNAs to ribosomes by recognizing mitochondrial tRNA T‐arms or D‐arms.^[^ ^13^ ^]^ Strikingly, besides the canonical role in translation, the bacterial EF‐Tu has various other functions during bacteria‐host interaction, such as immune response activation in Brassicaceae plants,^[^ ^7^ ^]^ host cell adherence,^[^ ^14^ ^]^ interaction with actin‐like cytoskeletal to regulate cell shape and biofilm formation^[^ ^15^ ^]^ and binding with host complement factors and plasminogen to inhibit host immune responses.^[^ ^16^ ^]^ According to the endosymbiotic theory, eukaryotic mitochondria are descended from alphaproteobacteria, wherein some mitochondrial protein‐coding genes are transferred from the endosymbiont to the host genome via endosymbiotic gene transfer.^[^ ^17^ ^]^ Hence, we hypothesize that the nematode EF‐Tu proteins are eubacterial in origin and could also have inherited some of its varied functions from its ancestor, like activation of host immunity. Here, we experimentally demonstrated that the *Meloidogyne javanica* EF‐Tu protein (MjEF‐Tu) indeed locates in mitochondria. At present, there are no reports indicating that mitochondrial proteins can be secreted into hosts by pathogens. In this study, we present evidence to show that *MjEF‐Tu* is upregulated in the nematode parasitic stage compared to the preparasitic second‐stage juvenile (pre‐J2) stage and mitochondrial‐localised MjEF‐Tu is secreted into host plants during nematode parasitism, sensed by the pattern‐recognition receptor AtEFR and eliciting plant hallmark defence responses. Moreover, an 18‐aa sequence (Nelf18) in the N terminus of nematode EF‐Tu contributes to the PAMP activity. Collectively, we experimentally demonstrate for the first time that mitochondrial proteins from pathogens can be secreted into hosts and function as a cross‐kingdom signal, and identified the first plant‐parasitic animal‐derived proteinaceous PAMP/PRR pair, providing novel insights into host‐pathogen interactions.

## 2 Results

### 2.1 Characterization of the *M. javanica* Gene MjEF‐Tu

Using *Meloidogyne* transcriptome data (ERP009887), we found that the expression of *EF‐Tu* was higher in the nematode parasitic stage compared to the pre‐J2 stage. Quantitative real‐time polymerase chain reaction (qRT‐PCR) confirmed that the *M. javanica EF‐Tu* gene (named *MjEF‐Tu*) is up‐regulated during nematode parasitism (Figure S1, Supporting Information). The full‐length cDNA sequence of *MjEF‐Tu* was obtained, including an open reading frame (ORF) of 1449 bp, encoding a 482‐aa polypeptide with a predicted molecular size of 54.2 kDa. It was predicted to contain a 16‐aa mitochondrial transit peptide (MTP) and three domains, that is, EF‐Tu GTP‐binding domain (IPR041709, 31–229 aa), EF‐Tu domain 2 (IPR033720, 250–318 aa) and EFTu/EF1A C‐terminal domain (IPR004160, 329–419 aa) (Figure S2A,B, Supporting Information). Protein sequence alignment analysis shows that the MjEF‐Tu shares 61%≈99.6% similarity with the EF‐Tu from other nematode species, the highest being between the MjEF‐Tu and the *M. incognita* EF‐Tu sequence (Figures S2C and S3, Supporting Information).

**Figure 1:**
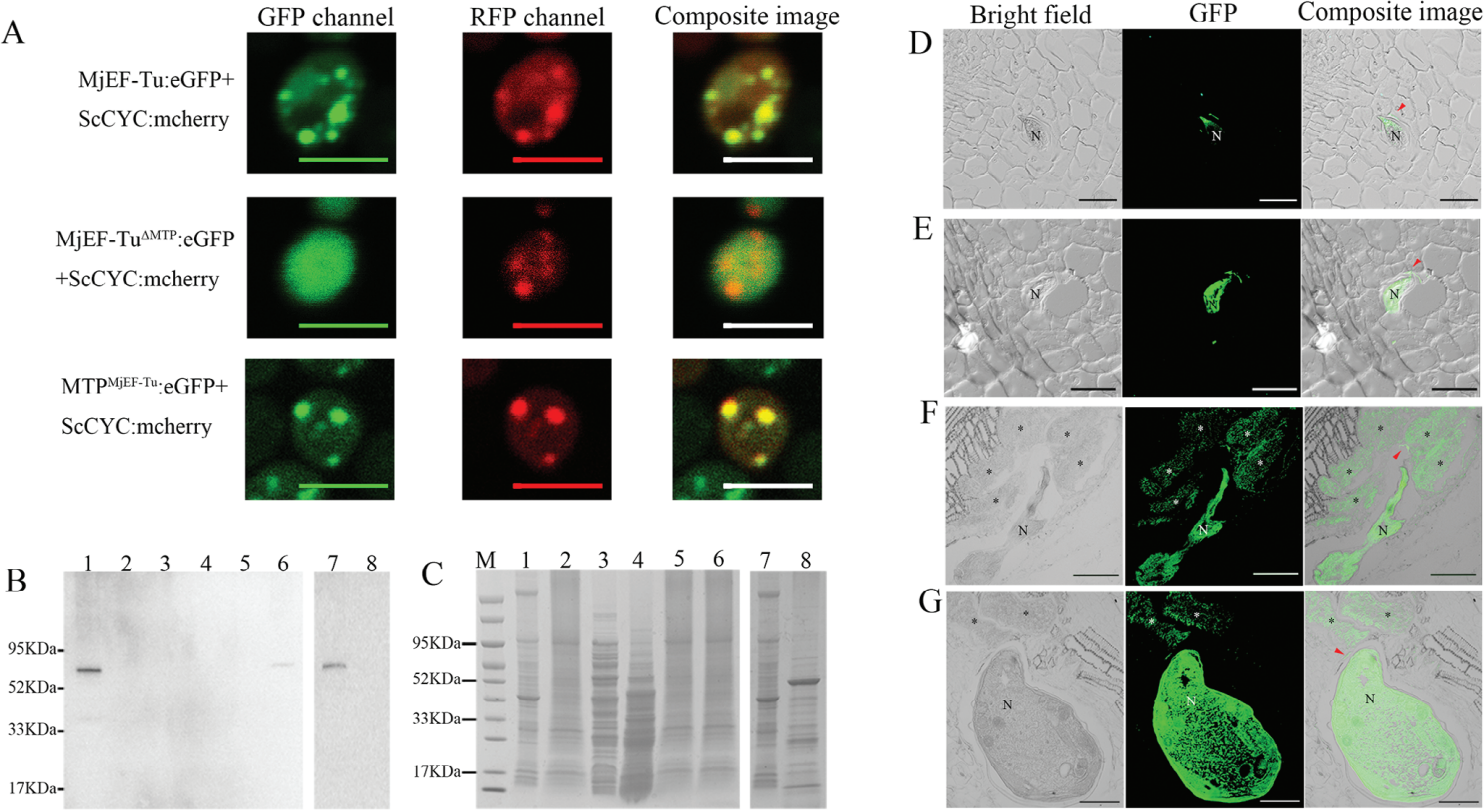
Mitochondrial protein MjEF‐Tu is secreted into host plants during parasitism of *Meloidogyne javanica*. A) MjEF‐Tu resides within the mitochondrion. *Saccharomyces cerevisiae* AH109 cells were cotransformed with MjEF‐Tu:eGFP/ScCYC:mcherry, MjEF‐Tu^ΔMTP^:eGFP/ScCYC:mcherry, MTP^MjEF‐Tu^:eGFP/ScCYC:mcherry; The images were taken at 48 h after cotransformation. eGFP, enhanced green fluorescent protein; mCherry, monomeric red fluorescent protein; MjEF‐Tu^ΔMTP^:eGFP, MjEF‐Tu protein lacking mitochondrial transit peptide (MTP) fused N‐terminally of eGFP; MTP^MjEF‐Tu^:eGFP, MTP of MjEF‐Tu fused N‐terminally of eGFP. ScCYC, a cytochrome c oxidase subunit IV protein of *Saccharomyces cerevisiae* located in mitochondria. Bars = 10 µm. B) Western blot analysis with anti‐MjEF‐Tu antibody of total proteins from the pre‐parasitic second‐stage juveniles (Lane 1 and Lane 7) of *M. javanica*, healthy tomato roots (Lane 2), *Ralstonia* sp. (Lane 3), *Saccharomyces* sp. (Lane 4) and tomato root sections without nematodes cut from tomato roots infected with *M. javanica* (Lane 5), tomato galls with *M. javanica* (Lane 6) and tomato leaves (Lane 8). C) Loading control of proteins stained with Coomassie brilliant blue; M: the protein's standard molecular weight. D‐G) MjEF‐Tu localisation in sectioned tomato root galls at 5 D,E), 10 F), and 18 dpi G). Red arrowhead showing MjEF‐Tu in the apoplast; N, nematode; *, giant cell; dpi, days post‐inoculation; Bars = 50 µm.

Due to the MjEF‐Tu with a MTP, heterologous protein expression was used to test whether it localizes in mitochondria. Enhanced green fluorescent protein (eGFP) was respectively fused to the C‐terminus of MjEF‐Tu (MjEF‐Tu:eGFP), MTP of MjEF‐Tu (MTP^MjEF‐Tu^:eGFP) and MjEF‐Tu without the MTP (MjEF‐Tu^ΔMTP^:eGFP). Meantime, red fluorescent protein (RFP) was fused to the C‐terminus of ScCYC (a cytochrome c oxidase subunit IV protein of *Saccharomyces cerevisiae* located in mitochondria) to generate the mitochondrial marker ScCYC:RFP. When MjEF‐Tu:eGFP, MTP^MjEF‐Tu^:eGFP or MjEF‐Tu^ΔMTP^:eGFP were expressed in *S. cerevisiae* together with ScCYC:RFP, the fusion proteins MjEF‐Tu:eGFP and MTP^MjEF‐Tu^:eGFP were detected to be colocalized in the mitochondria with ScCYC, but MjEF‐Tu^ΔMTP^:eGFP was not detected in the mitochondria (**Figure**  **1**A). Therefore, the MjEF‐Tu is indicated to be a mitochondrial protein, and the MTP of MjEF‐Tu functions in transferring the protein into mitochondria.

### 2.2 MjEF‐Tu is Secreted into Plants During Nematode Parasitism

Given the up‐regulation of *MjEF‐Tu* in parasitic stages of *M. javanica*, it may play roles in the pathogen‐host interaction. To achieve this, MjEF‐Tu needs to be released into hosts first, however mitochondrial proteins have not previously been shown to be secreted into hosts by pathogens. To examine whether MiEF‐Tu is secreted into host plants during nematode parasitism, *in planta* immunolocalization was conducted using an antibody specific to MjEF‐Tu. Western blot analysis was used to determine the antibody specificity to MjEF‐Tu, which showed a clear band with the expected ≈55 kDa size in the total protein samples from the pre‐J2s and tomato galls with nematodes but not in the protein samples from the healthy tomato roots and leaves, tomato root sections without nematodes cut from tomato roots infected with *M. javanica*, the bacteria *Ralstonia* sp. and the fungal *Saccharomyces* sp. isolated from *M. javanica* (Figure 1B,C; Supporting Results). Therefore, the anti‐MjEF‐Tu antibody could specifically recognise the MjEF‐Tu of *M. javanica*.

The localisation of the MjEF‐Tu protein was consistently observed along the cell wall near the nematode head at 5, 10, and 18 dpi (Figure 1D–G). At 10 dpi and 18 dpi, the signal of MjEF‐Tu was also observed within giant cells (Figure 1F,G). No signals were observed in the cells of either gall sections‐containing nematodes without antibody or in root sections of an uninfected, healthy plant, treated with the anti‐MjEF‐Tu antibody (Figure S4, Supporting Information). In sections, MjEF‐Tu was also observed in the whole nematode (Figure 1D–G). Accordingly, immunolocalization was also performed on the pre‐J2s. The result showed that MjEF‐Tu was ubiquitously present throughout the nematode (Figure S5A,B, Supporting Information), and no signal was observed in pre‐J2s incubated with the pre‐immune serum (Figure S5C,D, Supporting Information). These findings suggested that the intracellular ubiquitously expressed protein MjEF‐Tu can be secreted into plants during nematode parasitism.

### 2.3 MjEF‐Tu Induces Plant Innate Immunity Sensed by AtEFR

**Figure 2:**
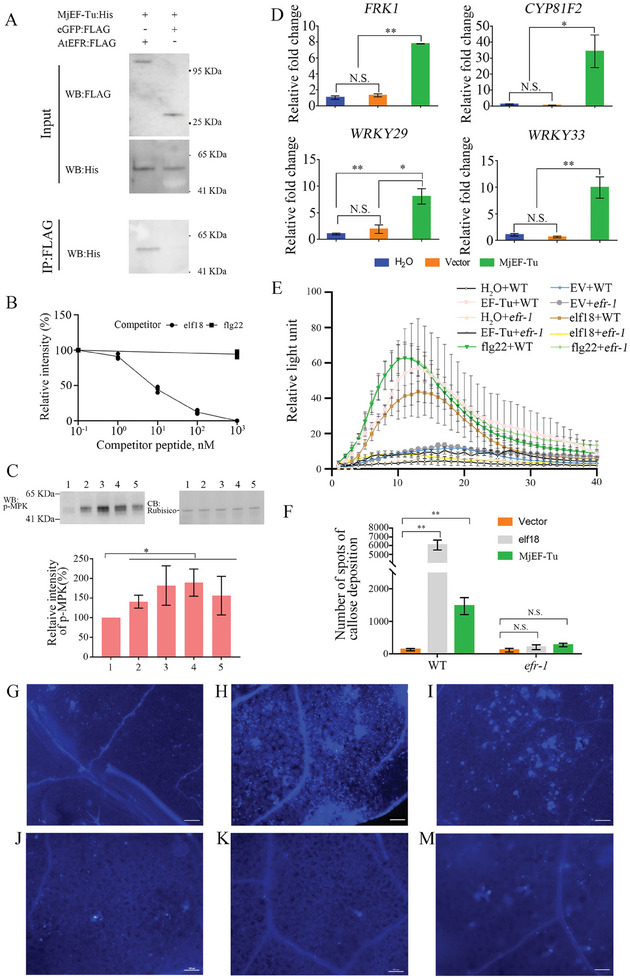
MjEF‐Tu induces plant innate immunity sensed by EFR. A) Coimmunoprecipitation analysis of MjEF‐Tu:His interacting with AtEFR:Flag. Western blot analysis confirmed the input proteins: eGFP:Flag, AtEFR:Flag and MjEF‐Tu:His; MjEF‐Tu:His was detected only after coimmunoprecipitation with the sample containing AtEFR:Flag but not eGFP:Flag; B) Competitive binding assays of MjEF‐Tu:His with different concentrations of unlabeled flg22 and elf18. Results are presented as relative intensity of MjEF‐Tu:His. Relative intensity was quantified by densitometry of MjEF‐Tu:His of 1, 10, 100, and 1000 nm unlabeled competitor treatment normalized to unlabeled 0.1 nM competitor treatment. Competitors were tested twice in independent assays. C) MAPKs activation in *A. thaliana* after treatment with different concentrations of MjEF‐Tu. CB, coomassie brilliant blue staining of total proteins (loading control); Relative band intensity of phosphorylated MPK6 in *A. thaliana*; 1, 2, 3, 4, and 5 represent treatment with 1 µg mL^−1^ protein purified from *Escherichia coli* with the empty vector, 1, 5, 10 and 30 µg mL^−1^ MjEF‐Tu, respectively; D) Relative expression fold change of defence genes *WRKY33*, *WRKY29*, *CYP81F2* and *FRK1* in Arabidopsis in response to MjEF‐Tu or protein purified from *E. coli* carrying pET28a vector compared to the treatment with H_2_O. The *A. thaliana* ubiquitin carboxyl‐terminal hydrolase 22 gene (AT5G10790) was used as an internal control and the relative fold change was relative to the expression of H_2_O treated plants. Values are presented as mean ± standard deviation (S.D.) of three biological replicates; E) MjEF‐Tu induces reactive oxygen species production in wild‐type (WT) Arabidopsis but not in Arabidopsis lacking the EF‐Tu receptor (*efr‐1*). The values are presented as the average of relative luminescence units ± S.D. of 8 leaf discs. H_2_O + WT, EF‐Tu + WT, EV + WT, elf18 + WT and flg22 + WT represent the WT Arabidopsis treated with H_2_O, MjEF‐Tu, protein purified from *E. coli* carrying pET28a vector, elf18 peptide, and flg22 peptide, respectively; H_2_O + *efr‐1*, EF‐Tu + *efr‐1*, EV + *efr‐1*, elf18 + *efr‐1* and flg22 + *efr‐1* represent Arabidopsis mutant *efr‐1* treated with H_2_O, MjEF‐Tu, protein purified from *E. coli* carrying pET28a vector, elf18 peptide, and flg22 peptide, respectively; F) The number of callose spots. Values are presented as mean ± S.D.; G–I) Callose deposition in WT Arabidopsis after treatment with protein purified from *E. coli* with the empty pET28a vector, elf18 peptide (elf18) and MjEF‐Tu (MjEF‐Tu) for 24 h; J–M) Callose deposition in *efr‐1* after treatment with protein purified from *E. coli* carrying pET28a vector, elf18 and MjEF‐Tu for 24 h. Callose deposition was analysed using aniline blue staining, and the number of fluorescent spots was determined using UV epifluorescence microscopy. **p < 0.01; N.S., no significant difference, Student's *t*‐test.

Considering the endosymbiosis theory and the fact that mitochondria‐localized MjEF‐Tu is secreted into the host cell apoplast, we hypothesized that MjEF‐Tu may interact with PRRs such as AtEFR,^[^ ^3^ ^]^ thereby regulating plant defense. Therefore, co‐immunoprecipitation (Co‐IP) assays were first performed to determine the interaction between AtEFR and MjEF‐Tu. AtEFR:Flag and eGFP:Flag were respectively expressed in *A. thaliana*, and MjEF‐Tu:His was expressed and purified from *E. coli* BL21. Western blot analysis was used to confirm the expression of the input proteins; bands were observed at ≈110, ≈28, and ≈58 KDa, matching the size of EFR:Flag, eGFP:Flag and MjEF‐Tu:His, respectively. Then, each sample was immunoprecipitated with Anti‐Flag‐Magnetic beads. The immunoprecipitated proteins were detected using an anti‐His antibody, and one specific band of ≈58 KDa was detected in the sample containing AtEFR:Flag and MjEF‐Tu:His, which is consistent with the size of MjEF‐Tu:His. As a control, no signals were detected using an anti‐His antibody in the sample containing eGFP:Flag and MjEF‐Tu:His (**Figure** **2**A). Additionally, competitive binding assays were performed to further determine the interaction. The relative signal intensity of MjEF‐Tu:His was decreased with the increased addition of elf18. The binding of AtEFR to MjEF‐Tu was completely inhibited by elf18 at a concentration of 1000 nm (Figure 2B; Figure S6, Supporting Information), showing that MjEF‐Tu and elf18 competitively interact with AtEFR. These findings indicate that MjEF‐Tu interacts with the PRR AtEFR.

Subsequently, typical events of PTI responses, including MAPK phosphorylation, ROS burst, defence‐related gene expression, and cell wall callose deposition,^[^ ^1^, ^18^, ^19^ ^]^ were evaluated in *Arabidopsis* with or without MjEF‐Tu treatment. It was found that 1–30 µg mL^−1^ of MjEF‐Tu could induce MAPK phosphorylation in wild type (WT) *Arabidopsis* (Figure 2C). Additionally, the qRT‐PCR analysis revealed that the transcript levels of several key PTI marker genes, *WRKY33*, *WRKY29*, *CYP81F2*, and *FRK1*,^[^ ^20^ ^]^ in WT *Arabidopsis* at 1 h after treatment with 10 µg mL^−1^ MjEF‐Tu were up‐regulated (Figure 2D). The ROS assay found that 10 µg mL^−1^ MjEF‐Tu obviously elicited ROS generation in WT *Arabidopsis* but not in *efr‐1* (Figure 2E). Similarly, cell wall callose deposition was significantly enhanced in WT *Arabidopsis* treated with MjEF‐Tu but not in *efr‐1* (Figure 2F–M).

Meantime, *Tobacco rattle virus* (TRV)‐mediated gene silencing was performed to knock down *AtEFR* by using RNA interference (RNAi) with the construct pTRV2:AtEFR. The qRT‐PCR analysis showed that the transcript abundance of *AtEFR* was drastically reduced in the plants infiltrated with the RNAi construct compared with the control construct (Figure S7A, Supporting Information), demonstrating the effectiveness of TRV‐mediated gene silencing. Significantly, MjEF‐Tu could induce MAPK phosphorylation in WT plants and plants infiltrated with the control construct, but not in the *AtEFR*‐silenced plants (Figure S7B,C, Supporting Information).

Due to the lack of *EFR* in Solanaceae plants,^[^ ^3^ ^]^ western blot analysis to detect MAPK phosphorylation in WT *Nicotiana benthamiana*, *N. benthamiana* expressing *AtEFR* and *N. benthamiana* expressing *eGFP* was performed. It was found that the phosphorylation level of MAPKs was obviously induced by MjEF‐Tu in *N. benthamiana* expressing *AtEFR*, but not in WT *N. benthamiana* and *N. benthamiana* expressing *eGFP* (Figure S8, Supporting Information). Hence, these results illustrate that the hallmark PTI responses can be activated by MjEF‐Tu and are sensed by AtEFR.

### 2.4 AtEFR Senses NemaWater and Mitochondrial Extracts

**Figure 3:**
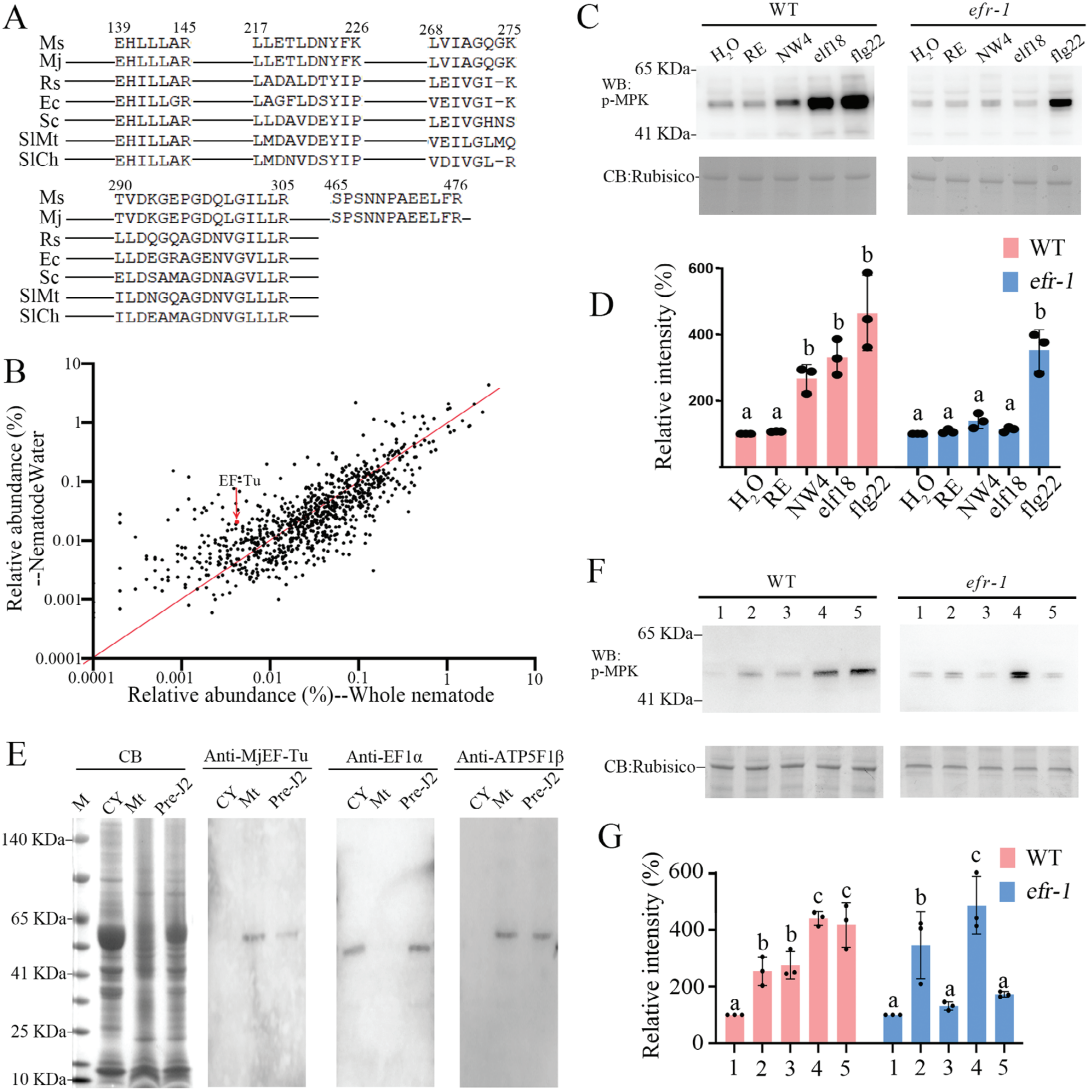
AtEFR senses nematode water extract (NemaWater) and mitochondrial extract. A,B) Mass spectrum analysis of MjEF‐Tu from NemaWater. A) Sequence alignment of EF‐Tu. Ms represents peptides of EF‐Tu identified from NemaWater. Mj, Rs, Ec, Sc, SlMt, and SlCh represent EF‐Tu from *Meloidogyne javanica*, *Ralstonia* sp., *E. coli*, *Saccharomyces cerevisiae*, mitochondria of *Solanum lycopersicum* and chloroplasts of *S. lycopersicum*, respectively; B) The relative abundance of each protein was calculated by intensity‐based absolute quantification. The ratio of relative abundance‐NemaWater to relative abundance‐total proteins of nematodes was calculated for each protein to evaluate whether the protein was enriched in the NemaWater; C,D) NemaWater activates MAPKs in *A. thaliana* sensed by AtEFR. Results are presented as relative intensity of p‐MPK. Relative intensity was quantified by densitometry of p‐MPK treated with NemaWater normalized to the treatment with H_2_O. Each value is the mean ± standard deviation of three replicates. H_2_O, RE, NW4, elf18, and flg22 represent treatment with H_2_O, 0.4% resorcinol, supernatant from *M. javanica* treated with 0.4% resorcinol for 4 h, elf18 and flg22 peptide, respectively. WT, wild‐type Arabidopsis; *efr‐1*, Arabidopsis lacking the EF‐Tu receptor (EFR). CB, coomassie brilliant blue staining of total proteins (loading control); Different letters indicate significant differences in the p‐MPK level among different treatments according to one‐way ANOVA followed by Duncan's multiple range tests (α = 0.05); E) Western blot analysis of MjEF‐Tu in the *M. javanica* mitochondrial extract. CY, Mt and Pre‐J2 represent the proteins extracted from the cytoplasm, mitochondria and preparasitic second‐stage juveniles of *M. javanica*, respectively; Anti‐MjEF‐Tu, the anti‐MjEF‐Tu antibody; Anti‐EF1α, the anti‐MjEF1α antibody; Anti‐ATP5F1β, the anti‐ATP5F1β antibody; F,G) Mitochondrial extracts of *M. javanica* activate MAPKs in *A. thaliana* sensed by AtEFR. Results are presented as relative intensity of p‐MPK. Relative intensity was quantified by densitometry of p‐MPK treated with different concentration of cytoplasmic or mitochondrial extract normalized to the treatment with H_2_O. Each value is the mean ± standard deviation of three replicates. Lanes 1, 2, 3, 4, and 5 represent treatment with H_2_O, 5 µg mL^−1^ cytoplasmic extract, 5 µg mL^−1^ mitochondrial extract, 50 µg mL^−1^ cytoplasmic extract, and 50 µg mL^−1^ mitochondrial extract for 10 min, respectively.

To further examine whether AtEFR can sense *M. javanica*‐derived MjEF‐Tu, we obtained the NemaWater extract of *M. javanica* pre‐J2s. Mass spectrum analysis revealed that peptides of EF‐Tu could be detected in NemaWater. The amino acid sequences of these peptides are the same as MjEF‐Tu but exhibit differences compared to the EF‐Tu sequences from bacteria and plants, indicating that they indeed derive from the MjEF‐Tu (**Figure** **3**A). The relative abundance of MjEF‐Tu in NemaWater compared to total proteins of nematodes showed that MjEF‐Tu is enriched in NemaWater (Figure 3B). Significantly, the phosphorylation level of MAPKs was clearly higher in WT *Arabidopsis* instead of in the *efr‐1* mutant when plants were treated with NemaWater extract of *M. javanica* pre‐J2s (Figure 3C,D).

Meantime, we obtained *M. javanica* mitochondrial extract and cytoplasmic extract. Western blot analysis using the anti‐MjEF‐Tu antibody revealed a clear band with the correct size of ≈55 kDa in the *M. javanica* mitochondrial extract and total protein. However, no signals were detected in the *M. javanica* cytoplasmic extract. For the controls, MjEF1α (50 kDa), a cytoplasm‐localised elongation factor, was detected using the anti‐EF1α serum in the cytoplasmic extract and total protein, but not in the mitochondrial extract of *M. javanica*. And MjATP5F1β (57 kDa), a soluble catalytic core β subunit of mitochondrial ATP synthase, was detected using an ATP5F1B monoclonal antibody in the mitochondrial extract and total protein, but not in the cytoplasmic extract of *M. javanica* (Figure 3E). The results confirm the presence of MjEF‐Tu in the mitochondrial extract, but not in the cytoplasmic extract. Interestingly, pre‐treatment with the *M. javanica* cytoplasmic extract significantly induces MAPKs phosphorylation not only in WT *Arabidopsis* but also in the *Arabidopsis* EFR mutant *efr‐1*. However, the phosphorylation level of MAPKs was clearly higher in WT *Arabidopsis* instead of in *efr‐1* when plants were treated with the mitochondrial extract (Figure 3F,G).

The above results demonstrate that AtEFR senses NemaWater and mitochondrial extracts triggering plant immunity, owing to the presence of MjEF‐Tu.

### 2.5 The Elicitor‐Active Epitope is Present in the N Terminus of Nematode‐Derived EF‐Tu

**Figure 4:**
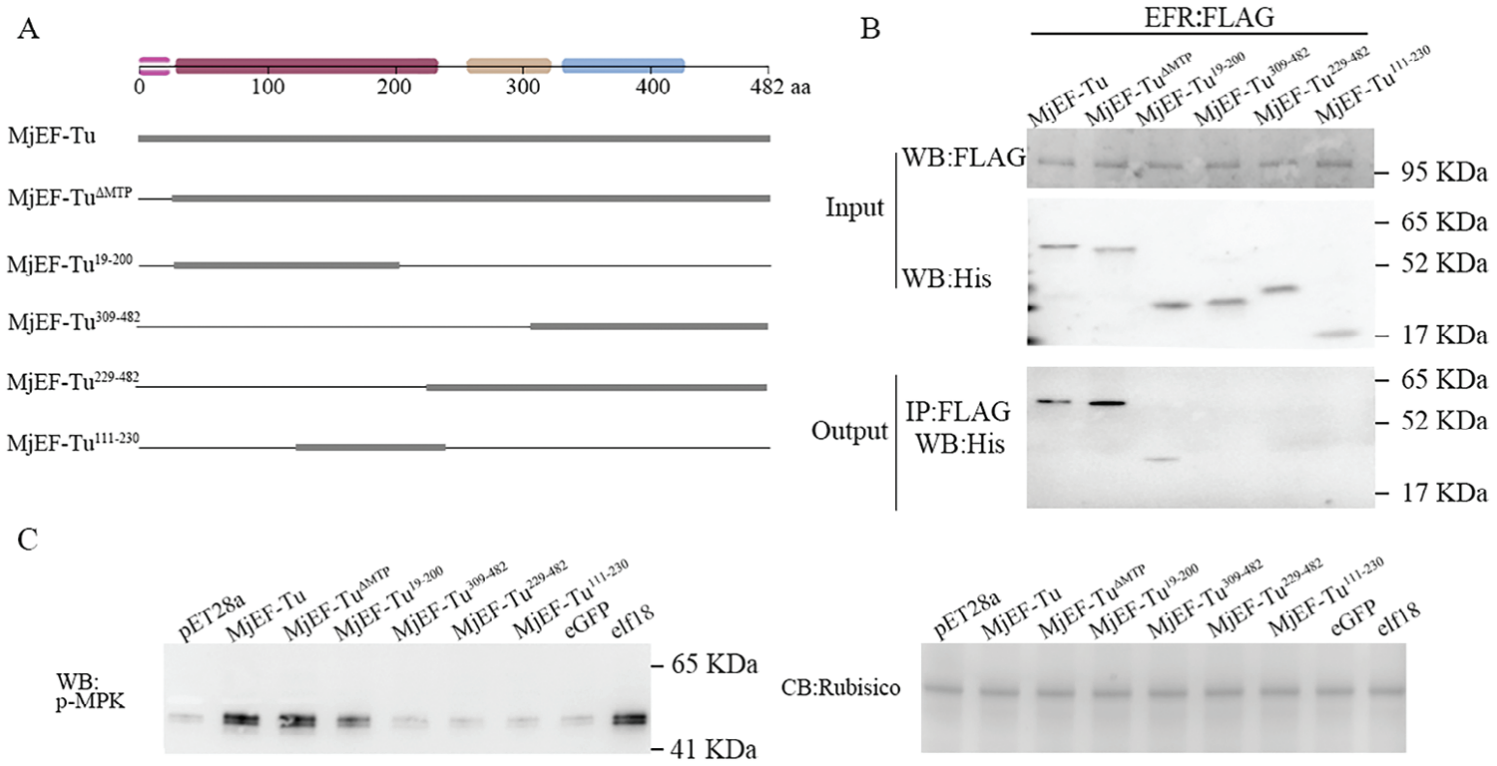
N‐terminus of MjEF‐Tu sensed by AtEFR. A) Schematic diagram showing the protein structures of MjEF‐Tu mutants; B) Western blot analysis confirmed the input proteins: AtEFR:Flag, MjEF‐Tu:His, MjEF‐Tu^ΔMTP^:His, MjEF‐Tu^19‐200^:His, MjEF‐Tu^229‐482^:His, MjEF‐Tu^309‐482^:His and MjEF‐Tu^111‐230^:His; only MjEF‐Tu:His, MjEF‐Tu^ΔMTP^:His, MjEF‐Tu^19‐200^:His were detected after coimmunoprecipitation; C) Phosphorylation‐inducing activity of truncated derivatives of MjEF‐Tu.

**Figure 5:**
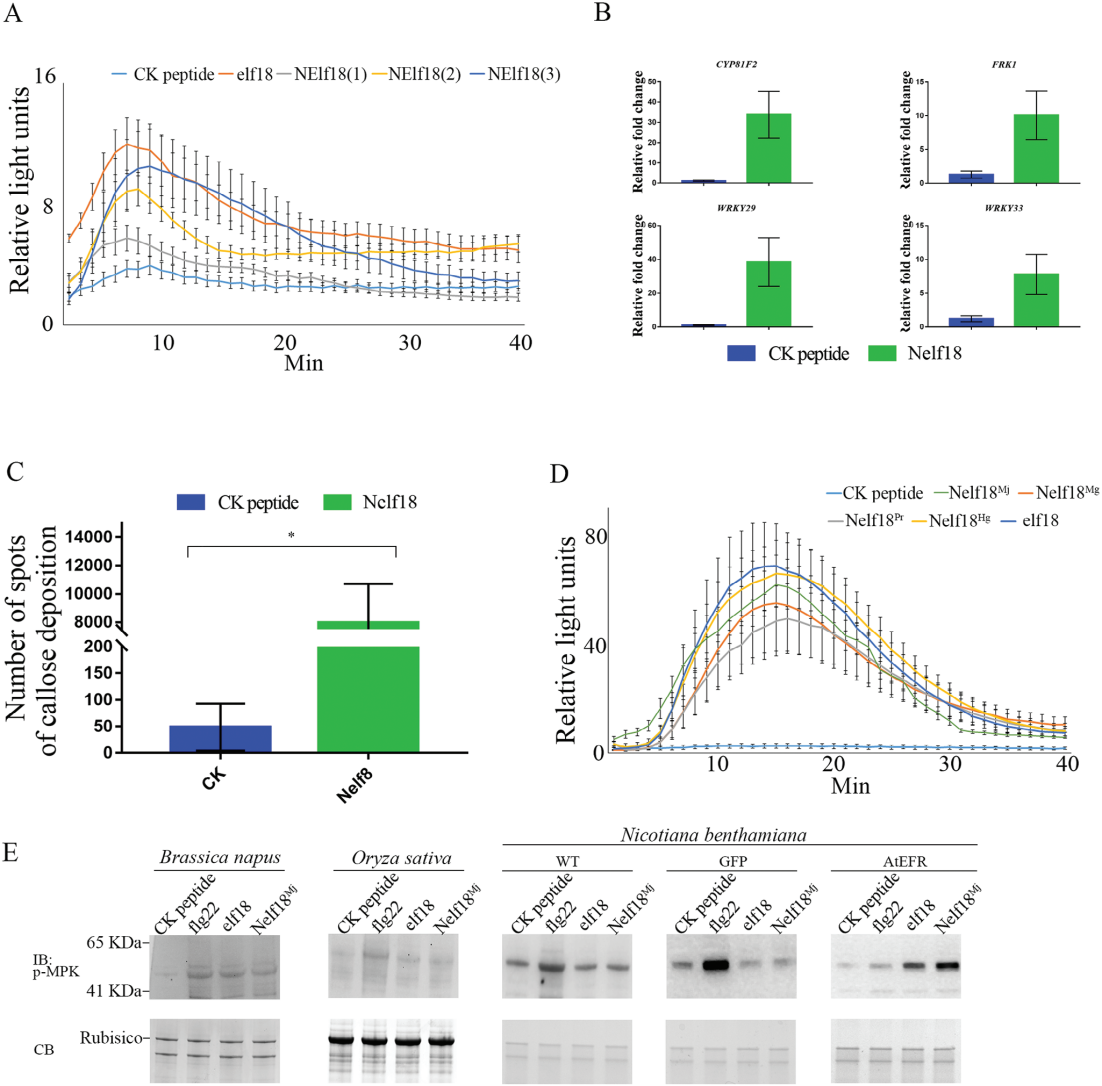
Induction of Nelf18 responses in different plant species. A) Different concentrations of *Meloidogyne javanica* Nelf18 peptide induce reactive oxygen species (ROS) production in Arabidopsis. The values are presented as the average of relative luminescence units (RLUs) ± standard deviation (S.D.) of 8 leaf discs. CK peptide, elf18, NElf18(1), NElf18(2) and NElf18(3) indicate treatment with 1 µM CK peptide, 1 µM elf18 peptide, 0.5 µM, 1 µM and 10 µM Nelf18 peptide, respectively; B) The relative expression fold change of defence genes *CYP81F*, *WRKY33*, *WRKY29*, and *FRK1* in Arabidopsis after treatment with *M. javanica* Nelf18 peptide (Nelf18) compared to plants treated with the CK peptide that is composed with random amino acids (SAHHAEHHHPHVHHAHHA). Values are presented as mean ± S.D. of three replicates; C) The number of callose deposition spots in *A. thaliana* after treatment with *M. javanica* Nelf18 peptide (Nelf18) and CK peptide (CK) for 24 h. Callose deposition was analysed using aniline blue staining, and the number of fluorescent spots was determined using UV epifluorescence microscopy. Values are presented as mean ± S.D.; D) Various Nelf18 peptides from different nematodes induce ROS production in Arabidopsis. The values of relative light units are presented as the average of RLUs ± S.D. of 8 leaf discs. *p < 0.05, Student's *t*‐test; CK peptide, elf18, Nelf18^Mj^, Nelf18^Mg^, Nelf18^Pr^, Nelf18 ^Hg^ and flg22 represent treatment with CK peptide, elf18 peptide, *M. javanica* Nelf18 peptide, *M. graminicola* Nelf18 peptide, *Pratylenchus brachyurus* Nelf18 peptide, *Heterodera glycines* Nelf18 peptide and flg22 peptide, respectively. E) Activation of MAPKs in *Brassica napus*, *Nicotiana benthamiana* and *Oryza sativa* after treatment with Nelf18. WT, eGFP and AtEFR represent wild‐type *N. benthamiana*, *N. benthamiana* expressing *eGFP* and *N. benthamiana* expressing *AtEFR*.

To examine which region has the PTI potential, a range of truncated derivatives of MjEF‐Tu were generated (**Figure** **4**A) based on the predicted structure of MjEF‐Tu (Figure S2A,B, Supporting Information). We first generated and expressed MjEF‐Tu^ΔMTP^. Subsequently, we divided MjEF‐Tu into four fragments, that is, a 19‐200‐aa fragment (MjEF‐Tu^19‐200^) containing the N‐terminal sequence and domain 1, a 111‐230‐aa fragment (MjEF‐Tu^111‐230^) containing part of domain 1 and domain 2, a 229‐482‐aa fragment (MjEF‐Tu^229‐482^) containing part of domain 2, domain 3 and the C‐terminal sequence, and a 309‐482‐aa fragment (MjEF‐Tu^309‐482^) containing domain 3 and the C‐terminal sequence (Figure 4A). Co‐IP assays showed that only MjEF‐Tu^ΔMTP^ and MjEF‐Tu^19‐200^ could interact with AtEFR (Figure 4B). Consistent with the Co‐IP results (Figure 4B), MjEF‐Tu^ΔMTP^ and MjEF‐Tu^19‐200^ elicited MAPK phosphorylation, while MjEF‐Tu^111‐230^, MjEF‐Tu^229‐482^ and MjEF‐Tu^309‐482^ did not elicit MAPK phosphorylation (Figure 4C). The above results suggested that the 19‐110‐aa fragment is involved in interacting with AtEFR and eliciting an immune response in *Arabidopsis*. Sequence analysis showed that the 23‐40‐aa region within MjEF‐Tu is highly conserved in the nematode EF‐Tu proteins and shares 61%–78% similarity to elf18, a well‐known elicitor‐active epitope of the bacterial EF‐Tu^[^ ^7^ ^]^ (Figure S9, Supporting Information). Consequently, this sequence was aptly named Nelf18 (Nematode elf18). Therefore, PTI assays were performed to analyse the inducing potential of Nelf18 peptides from different PPNs. The Nelf18 peptide from *M. javanica* was found to activate hallmark immune responses in the Brassicaceae plant *Arabidopsis thaliana*, such as ROS burst, PTI marker gene up‐regulation and callose deposition (**Figure** **5**A–C). Further, Nelf18 peptides from *M. graminicola*, *Pratylenchus brachyurus* and *H. glycines* were shown to trigger a burst of ROS in *A. thaliana*, similar to the *M. javanica* Nelf18 (Figure 5D), suggesting that the elicitor‐active epitope resides in the N terminus of nematode‐derived EF‐Tu.

To further assess the potential of Nelf18 to induce immune responses in plants of different families, MAPK phosphorylation was examined in the Brassicaceae plant *Brassica napus*, the Solanaceae plant *N. benthamiana* and the Gramineae plant *Oryza sativa* with or without treatment of *M. javanica* Nelf18 peptide and two control PAMPs, flg22 and elf18. It is found that MAPK phosphorylation was induced with treatment of *M. javanica* Nelf18, flg22 and elf18 in *B. napus*. In *O. sativa* and *N. benthamiana*, MAPK phosphorylation was induced only by flg22, not by *M. javanica* Nelf18 and elf18. However, when *AtEFR* was expressed in *N. benthamiana*, MAPK phosphorylation was significantly induced with treatment of *M. javanica* Nelf18 and elf18 (Figure 5E). These results show that only Brassicaceae plants can sense nematode‐derived EF‐Tu.

### 2.6 Nelf18 Enhances Plant Resistance to Plant Pathogens Depending on EFR

**Figure 6:**
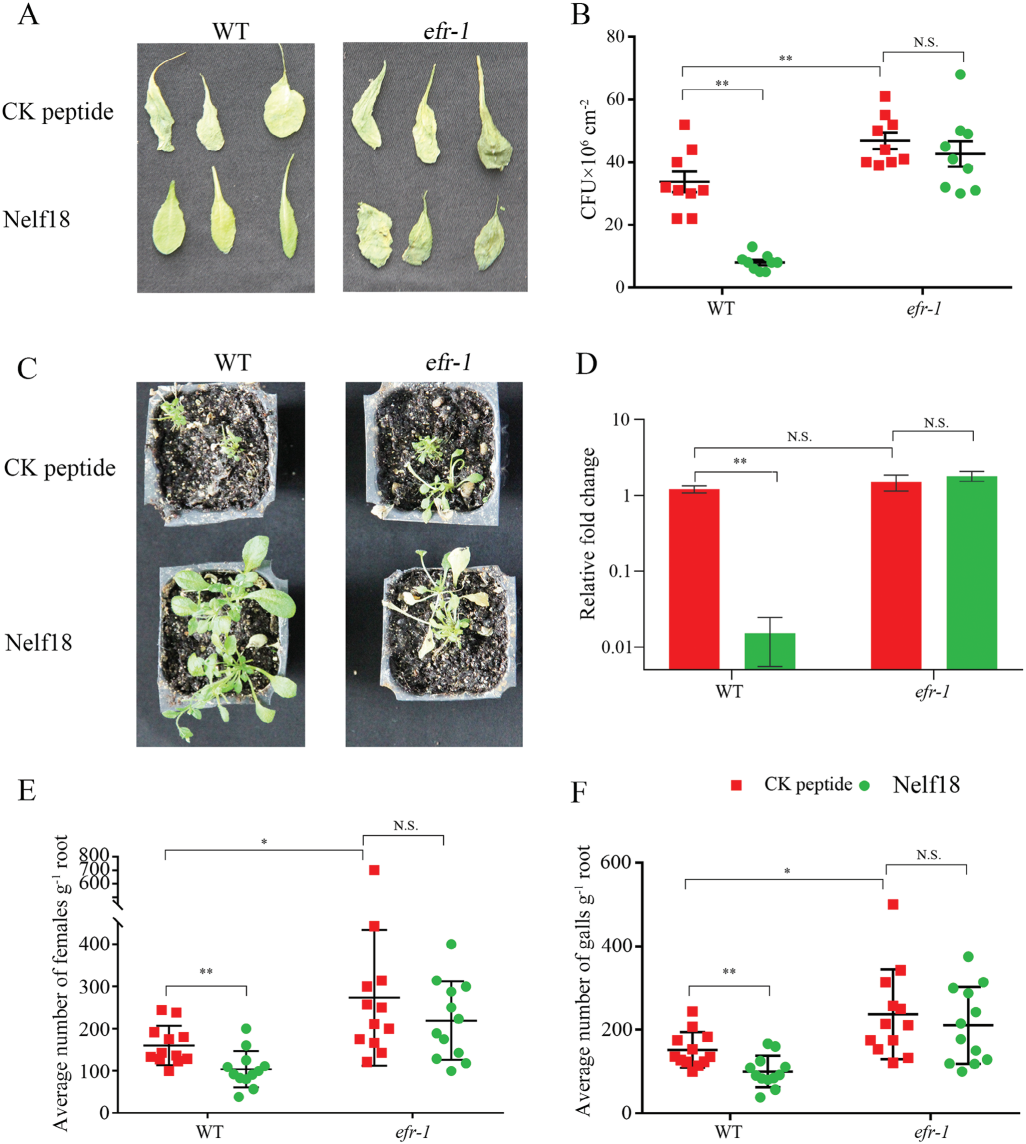
Nelf18 enhances resistance of Arabidopsis to plant pathogens. A, B) Symptoms and colony‐forming unit (CFU) of wild‐type (WT) Arabidopsis and Arabidopsis lacking EF‐Tu receptor (*efr‐1*) at day 4 after pressure infiltration with *Pst* DC3000. Leaves were pre‐treated with CK peptide that is composed with random amino acids (SAHHAEHHHPHVHHAHHA) or *M. javanica* Nelf18 peptide for 24 h. Data are presented as mean ± standard deviation; C) Symptoms on CMV‐inoculated plants; D) Reverse transcription quantitative PCR analysis of CMV *coat protein* (*CP*) accumulation in CMV‐inoculated Arabidopsis; E, F) The effect of *M. javanica* Nelf8 on Arabidopsis susceptibility to *M. javanica*. WT Arabidopsis and *efr‐1* were pretreated with CK peptide or *M. javanica* Nelf18 for 24 h before inoculation with ≈200 freshly hatched juveniles per plant. The number of females (E) and galls (F) were counted at 4 weeks after inoculation. The data are presented as the number of females or galls divided by the weight of the root. *p < 0.05, **p < 0.01; N.S., no significant difference, Student's *t*‐test.

Finally, we wanted to assess if the PTI responses triggered by Nelf18 can enhance plant defence against pathogens. To answer this, *Arabidopsis* leaves were treated with *M. javanica* Nelf18 for 24 h before inoculation with *Pseudomonas syringae* pv. tomato DC3000 (Pst DC3000) and *cucumber mosaic virus* (CMV), respectively. After 4 days, the growth of *Pst* DC3000 and the disease symptom of leaf curling/crinkling were obviously reduced in WT *Arabidopsis* with Nelf18 treatment compared to WT *Arabidopsis* with CK peptide treatment (**Figure** **6**A,B). After 30 days, the transcripts of CMV *coat protein* (*CP*) and the disease symptoms of leaf curling/crinkling and inflorescence underdevelopment were significantly reduced in WT Arabidopsis treated with Nelf18 compared to control plants (Figure 6C,D). Similarly, root drenching with Nelf18 provided strong protection against *M. javanica* infection as indicated by ≈55% fewer females and ≈60% fewer galls in WT *Arabidopsis* treated with Nelf18 than that in the control plants (Figure 6E,F). Conversely, Nelf18 did not enhance resistance to these three pathogens in *efr‐1* (Figure 6), indicating that Nelf18 could enhance *Arabidopsis* resistance against three major classes of pathogens, depending on EFR.

## 3 Discussion

Plant PRRs sense PAMPs to activate PTI against pathogens, which is an important plant defence mechanism.^[^ ^21^ ^]^ Recently, increasing numbers of microbe‐derived PAMPs and their cognate PRRs were discovered, with a few PAMPs being reported to exist across various microbial kingdoms.^[^ ^22^, ^23^, ^24^ ^]^ So far, no plant‐parasitic animal (including nematode)‐derived proteinaceous PAMP‐PRR pairs have been reported. In this study, the nematode‐derived proteinaceous PAMP MjEF‐Tu, a mitochondria‐localised translation elongation factor, and its cognate PRR AtEFR were identified.

Based on the endosymbiotic theory, the nematode EF‐Tu originates from alphaproteobacterium via endosymbiotic gene transfer.^[^ ^17^ ^]^ Transcriptional level analysis in our study indicated that the nematode *EF‐Tu* has a higher expression in nematode parasitic stages compared to the pre‐J2 stage. In silico analysis showed that the nematode‐derived EF‐Tu proteins have a conserved 18‐aa sequence at the N‐terminus (Nelf18) similar to elf18. Thus, we hypothesized that the nematode‐derived EF‐Tu may function as a PAMP to stimulate plant innate immunity. As immunogenic patterns are sensed by host cell surface receptors, release of PAMPs to host cells is a key premise.^[^ ^25^ ^]^ For microbes, small quantities of intracellular components could be released during the regular growth, or from lysed cells, or through outer membrane vesicles endocytosis into host cells.^[^ ^7^, ^26^, ^27^ ^]^ Hypothetically, the bacterial EF‐Tu is exported to host tissues via membrane vesicle endocytosis or cell lysis.^[^ ^7^, ^27^ ^]^ Previously, MiEF‐Tu, an orthologous MjEF‐Tu protein from *M. incognita*, was directly identified using nanoLC ESI MS/MS in NemaWater of pre‐J2s after treatment with neuroregulators,^[^ ^28^ ^]^ which doesn't mean MiEF‐Tu must be released into host plants in nematode parasitic stages. In this study, it was found that MjEF‐Tu is a mitochondria‐localised protein. To our knowledge, so far no mitochondria‐localised protein has been indicated to be secreted into hosts by pathogens. Therefore, it was crucial to determine whether EF‐Tu from the nematode gets out of the nematode and is secreted into hosts during the parasitic stages. Accordingly, *in planta* immunolocalisation was conducted using an antibody against MjEF‐Tu in this study and the results clearly showed that the mitochondria‐localised MjEF‐Tu could be secreted into the host plant during nematode parasitism.

Further experiments in this study revealed that the MjEF‐Tu protein can trigger PTI responses in Arabidopsis. Several corresponding Nelf18 peptides from different PPN also activated immune responses of Arabidopsis, such as ROS burst, PTI marker gene up‐regulation and callose deposition, and enhanced Arabidopsis resistance to *Pst* DC3000, CMV and *M. javani*ca. Previous studies have revealed that the EFR of Arabidopsis recognizes the bacterial PAMP EF‐Tu and its derived peptide, elf18.^[^ ^3^ ^]^ In this study, we found that MjEF‐Tu can interact with AtEFR. Moreover, the Arabidopsis mutant lacking EFR (*efr‐1*) was treated with MjEF‐Tu, Nelf18, *M. javanica* NemaWater and mitochondrial extracts, wherein the immune responses were weaker in *efr‐1* compared with that in WT Arabidopsis. The immune responses were also weaker in *EFR*‐silenced Arabidopsis when using MjEF‐Tu to treat plants. Our further analysis found that the *M. javanica* Nelf18 can induce MAPK phosphorylation in *B. napus*, but not in *N. benthamiana* and *O. sativa*. Not only MjEF‐Tu but also Nelf18 could induce MAPK phosphorylation in *N. benthamiana* expressing AtEFR. Thus, the nematode EF‐Tu is recognized by Brassicaceae plants, with this recognition requiring EFR, thereby inducing PTI. Interestingly, some other pathogen proteins have been reported to act as a PAMP but also as an important virulence factor during pathogen infection, such as flagellin from *Pseudomonas syringae* ^[^ ^6^, ^29^ ^]^ and XEG1 from *Phytophthora sojae*.^[^ ^30^ ^]^ In this study, *in planta* immunolocalization demonstrated that MjEF‐Tu is secreted into not only the host apoplast but also the giant cells, suggesting that in addition to its role in activating plant basal immunity, MjEF‐Tu may also perform other functions, such as contributing to the parasitism of the root‐knot nematode, which remains a topic for further study.

Mitochondria are crucial for the survival of eukaryotic cells because they play key roles in biological energy, metabolism and signalling.^[^ ^31^ ^]^ There are ≈1000 proteins located in the mitochondria to perform various roles.^[^ ^32^ ^]^ Notably, the intracellular mitochondrial proteins are transported to the apoplast and participate in the trigger of autoimmune responses as endogenous signals in animals,^[^ ^33^ ^]^ for example, the concentration of mitochondrial proteins in the human blood is a positive association of autoimmune diseases.^[^ ^34^ ^]^ Our study clearly shows that the *M. javanica* mitochondrial protein MjEF‐Tu is secreted into the host during nematode parasitism. It is generally accepted that PPN secreted proteins come from nematode secretory organs, including oesophageal gland cells, amphids and cuticles.^[^ ^35^ ^]^ In this study, it is found that the mitochondrial protein MjEF‐Tu was expressed in the whole nematode. We noticed that recent research exhibited that intracellular mitochondrial proteins can be actively packaged to extracellular vesicles (EVs) and secreted extracellularly,^[^ ^36^ ^]^ and EVs of the free‐living nematode *C. elegans* were shown to be produced in amphids of the head and secreted into the environment.^[^ ^37^ ^]^ Therefore, whether the intracellular ubiquitously expressed mitochondrial protein EF‐Tu of nematodes is secreted into plant cells through EVs, requires further investigation. No matter how it is released, *in planta* immunolocalisation in this study clearly showed that MjEF‐Tu is secreted into the host plant during nematode parasitism. Moreover, MjEF‐Tu elicits plant basal immunity sensed by the pattern‐recognition receptor AtEFR. These findings first demonstrate that mitochondrial proteins of pathogens can be secreted into hosts and function as cross‐kingdom molecules, which is likely universal in pathogen infection, highlighting the role of pathogen mitochondria during pathogen‐host interactions. Concurrently, this study also identified the first plant‐parasitic animal‐derived proteinaceous PAMP/PRR pair, providing novel insights into host‐pathogen interactions.

## 4 Conclusion

In summary, our study demonstrates that the *Meloidogyne javanica* EF‐Tu (MjEF‐Tu) resides in the nematode mitochondria and is released into the host plant during nematode parasitism. MjEF‐Tu interacts with the PRR AtEFR, triggering the plant hallmark defence responses mediated by AtEFR. And an 18‐aa sequence (Nelf18) in the N terminus of the nematode EF‐Tu contributes to the immunogenic activity. These findings strongly suggest that mitochondrial proteins from pathogens can be secreted into hosts and function as a cross‐kingdom signal, and MjEF‐Tu/AtEFR is the first plant‐parasitic animal‐derived proteinaceous PAMP/PRR pair, providing novel insights into host‐pathogen interactions.

## 5 Experimental Section

### Nematode and Plant Materials

*M. javanica* was collected from a towel gourd (*Luffa* sp.) in Guangxi, China, using a single egg mass, and maintained on tomato plants (*Solanum lycopersicum*) in a greenhouse at 25 °C under 16 h light/8 h dark conditions. Egg masses, pre‐J2s, and parasitic stage nematodes were collected as previously described.^[^ ^38^ ^]^ The EF‐Tu receptor Arabidopsis T‐DNA mutant line (SALK_044334, *efr‐1*)^[^ ^3^ ^]^ was obtained from the Arabidopsis Information Resource and confirmed by the MAPK activation assay in response to elf18 (Figure S10, Supporting Information). *A. thaliana*, *N. benthamiana* and *O. sativa* were cultivated at 25 °C, and *B. napus* at 20 °C, under 16 h light/8 h dark in a glasshouse at Zengcheng campus teaching & research base of South China Agricultural University

### Synthesis of Peptides and Primers

Peptides (Table S1, Supporting Information) were synthesized using a PepPower system (Genscript) and dissolved in water. Primers (Table S2, Supporting Information) were synthesised by Generay (Generay Biotech) and dissolved, diluted to 10 mm using water.

### Preparation of Nematode Cytoplasmic and Mitochondrial Extracts

Cytoplasmic and mitochondrial extracts were prepared from the pre‐J2s of *M. javanica* using Mitochondria/cytosol Fractionation Kit (Beyotime Biotechnology). Approximately 50000 nematodes were collected and washed twice with 500 µL of reagent A in a 1.5 mL tube. A total of 200 µL reagent A was added into the tube and nematodes were ground on ice. Following this, the mixture was centrifuged at 1000 g, 4 °C for 5 min. Subsequently, the supernatant was transferred to a new 1.5 mL tube and centrifuged at 11 000 g, 4 °C for 5 min. The supernatant contained the cytoplasmic extracts, whereas the pellets were washed using 500 µL reagent A and dissolved in 100 µL mitochondrial lysate buffer to obtain the mitochondrial extracts.

### Gene Amplification and Sequence Analyses

Total RNA of *M. javanica* was isolated using RNAprep Pure Micro Kit (TianGen Biotech). The full‐length cDNA sequence of *MjEF‐Tu* was amplified using the primer pair MjEF‐TuF/MjEF‐TuR. The amplification program was as follows: 94 °C for 30 s, 30 cycles of 94 °C for 10 s, 58 °C for 30 s, and 68 °C for 1 min. The homologous sequences of MjEF‐Tu were obtained from the National Center for Biotechnology Information and WormBase. The mitochondrial transit peptide was predicted using TargetP‐2.0^[^ ^39^ ^]^ and the sequence logo was generated using WebLogo.^[^ ^40^ ^]^ Further, conserved domains were analyzed using InterPro.^[^ ^41^ ^]^ The EF‐Tu proteins were aligned using the ClustalW method via MEGA‐X software.^[^ ^42^ ^]^

### Developmental Expression Analysis

The developmental expression analysis was performed using quantitative real‐time PCR (RT–qPCR), as described previously.^[^ ^43^ ^]^ Briefly, RNA samples were prepared from different life stages of *M. javanica*, using the RNAprep Pure Micro kit (Tiangen Biotech, Beijing, China). The cDNA was synthesized using TransScript One‐Step gDNA Removal and the cDNA Synthesis SuperMix kits (AT311, TransGen Biotech, Beijing, China). RT‐qPCR was performed using the primer pairs qMjEFTuF/qMjEFTuR and qMjActinF/qMjActinR for amplifying the gene *MjEF‐Tu* and the internal control gene *Mj‐β‐actin* (accession no. AF532605), respectively. RT‐qPCR was performed using the Green qPCR SuperMix (TransGen Biotech, Beijing, China). The relative changes in gene expression were determined using the 2^−ΔΔCT^ method.^[^ ^44^ ^]^ These experiments were repeated two times, with three technical replicates for each reaction.

### Mutagenesis and Purification of MjEF‐Tu Proteins

The sequences of MjEF‐Tu, MjEF‐TU^ΔMTP^, MjEF‐TU,^19‐200^ MjEF‐TU^229‐482^, MjEF‐TU^309‐482^, MjEF‐TU^111‐230^ and eGFP were respectively cloned into the pET28a expression vector to generate recombinant protein expression constructs, that is, pET28a:MjEF‐Tu:His, pET28a:MjEF‐Tu^ΔMTP^:His, pET28a: MjEF‐TU^19‐200^:His, pET28a:MjEF‐TU^229‐482^:His, pET28a:MjEF‐TU^309‐482^:His, pET28a: MjEF‐TU^111‐230^:His and pET28a:eGFP:His. These recombinant proteins were produced in *E. coli* BL21 (DE3) cells and purified using Ni^2+^NTA agarose, following the manufacturer's instruction (Qiagen). The amount of purified protein was determined using the Bradford method (Beyotime Biotechnology), whereas purity was analysed using sodium dodecyl sulphate‐polyacrylamide gel electrophoresis (SDS‐PAGE) (Figure S11, Supporting Information).

### Anti‐MjEF‐Tu Polyclonal Antibody Production and Immunofluorescence Localisation

Anti‐MjEF‐Tu polyclonal antibody was obtained as previously described.^[^ ^45^ ^]^ Briefly, the purified MjEF‐Tu protein was used to immunize rabbits intradermally for antiserum production.^[^ ^45^ ^]^ Anti‐MjEF‐Tu polyclonal antibody was purified from the antiserum using MjEF‐Tu conjugated agarose. The specificity of the anti‐MjEF‐Tu polyclonal antibody was determined using western blot analysis (Table **1**). For western blots, 10 µg of total proteins from pre‐J2s, galls cut from tomato roots infected with *M. javanica* at 10 days post‐inoculation, healthy tomato roots and leaves, and tomato root sections without nematodes cut from tomato roots infected with *M. javanica*, the bacteria *Ralstonia* sp. and the fungal *Saccharomycetales* sp. isolated from *M. javanica* (Supporting Methods and Results) were separated on SDS‐PAGE gel and transferred to a nitrocellulose membrane (PALL, Washington, NY, U.S.A.). The membranes were blocked with 5% (w/v) non‐fat milk and incubated with anti‐MjEF‐Tu antibody and soaked in the anti‐rabbit horseradish peroxidase‐conjugated secondary anti‐body (Cell Signaling Technology, Inc, U.S.A.) (Table 1), and the membranes were developed as described previously.^[^ ^46^ ^]^ For immunolocalization on gall sections, tomato roots infected with *M. javanica* for 5, 10, and 18 days were dissected, fixed, dehydrated, and embedded in paraffin as previously described,^[^ ^46^ ^]^ respectively. Sections were incubated in dimethyl benzene and an alcohol gradient to remove the paraffin and were subsequently treated with an Anti‐MjEF‐Tu primary antibody (Table 1) at room temperature for 4 h in a humid box. The sections were washed thrice for 5 min using phosphate‐buffered saline and then incubated with goat anti‐rabbit super clonal secondary antibody, Alexa Fluor 488 conjugate (Thermo Fisher Scientific) (Table 1) at room temperature for 2 h in a humid box. Finally, the sections were mounted with Fluoromount‐G (SouthernBiotech) and observed under a Nikon ECLIPSE Ni microscope. For immunolocalization on pre‐J2s of *M. javanica*, ≈10 000 freshly hatched pre‐J2s were used. Immunolocalization was carried out as described previously.^[^ ^47^ ^]^

**Table 1:** Antibodies.

Reagent/Resourc	Reference or Source	Identifier
Phospho‐p44/42 MAPK (erk1/2) (Thr202/Tyr204)	Cell Signaling	9101
Goat Anti‐Rabbit Immunoglobulins/HRP	Cell Signaling	7074P2
Goat Anti‐Rabbit Immunoglobulins/Alexa Fluor 488	TransGen Biotech	HS131‐01
Goat Anti‐Mouse Immunoglobulins/HRP	TransGen Biotech	HS201‐01
Anti‐His/HRP	Beyotime Biotechnology	AF2873
Anti‐DDDDK/HRP (Anti‐Flag/HRP)	ABclonal	AE024
Anti‐ATP5F1B	Beyotime Biotechnology	AG1190
Anti‐EF1α	This study	**/**
Anti‐MjEF‐Tu	This study	**/**

### Subcellular Localization of MjEF‐Tu in Yeast

First, the coding sequences of *eGFP* and *monomeric red fluorescent protein*  (mCherry) were amplified using the primer pairs BKGFPF/BKGFPR and ADRFPF/ADRFPR1, respectively. Second, the GAL4BD of pGBK vector and GAL4AD of pGAD vector were replaced by *eGFP* and *mCherry* sequences to generate the vectors pKGF and pARF, respectively. Thirdly, the coding sequences of *MjEF‐Tu* and *MjEF‐Tu* ^ΔMTP^ were amplified using primer pairs BKELFF/BKELFR and BKELF‐mpF/BKELFR, respectively. And the MjEF‐Tu MTP was synthesised by Generay (Generay Biotech). These sequences were cloned into pKGF vector to generate pKGF:MjEF‐Tu:eGFP, pKGF:MjEF‐Tu^ΔMTP^:eGFP, and pKGF:MTP^MjEF‐Tu^:eGFP, respectively. In addition, the coding sequence of the mitochondrial gene *ScCYC* was amplified using primer pair ARScF/ARScR and cloned into pARF to generate pARF:ScCOX:RFP as marker. Subsequently, the resulting pKGF vectors were separately co‐transformed with pARF:ScCOX:RFP into the yeast strain AH109 and the transformed yeast cells were growth on SD/‐Trp‐Leu media. Finally, the cells were observed under a Nikon ECLIPSE T*i* confocal system.

### Co‐IP Assays

The coding sequences of *AtEFR* and *eGFP* fused with a FLAG‐tag at the C‐terminus were cloned into the pCAMBIA1305 vector to generate p1305:AtEFR:Flag and p1305:eGFP:Flag. These vectors were expressed in Arabidopsis through *Agrobacterium tumefaciens* EHA105. At 48 h after Agro‐infiltration, the proteins were isolated from 1 g leaves using GTEN buffer.^[^ ^43^ ^]^ Immunoprecipitation was performed as follows: 1 µg MjEF‐Tu:His or its truncated derivatives and 20 µL Anti‐Flag Magnetic Beads were added to the proteins and incubated at 7 °C for 8 h on a rotator, the beads were collected and washed four times with GTEN buffer. Subsequently, the beads were resuspended in SDS‐PAGE loading buffer. The associated proteins were detected with an anti‐FLAG and an anti‐His antibody (Table 1), respectively, by western blot.

### Competitive Binding Assay

The competitive binding assay was performed as described previously.^[^ ^3^, ^48^ ^]^ Briefly, magnetic beads binding AtEFR:Flag and MjEF‐Tu:His were resuspended in GTEN buffer with 0.1, 1, 10, 100, and 1000 nm of elf18 peptide, respectively. For the control, magnetic beads binding AtEFR:Flag and MjEF‐Tu:His were resuspended in GTEN buffer with 0.1 and 1000 nm of flg22 peptide, respectively. These mixtures were then incubated at 7 °C for 1 h on a rotator, after which the beads were collected and washed four times with GTEN buffer. The immunodetected MjEF‐Tu:His bands were quantified using the ImageJ software.

### TRV‐Mediated AtEFR Silencing

A 360 bp fragment of the *AtEFR* gene was amplified by PCR using the primer pair TRV‐efrF/TRV‐efrR. The fragment was cloned into the pTRV2 vector digested with *Xba*I and *Kpn*I for generating pTRV2:AtEFR. The vectors pTRV1, pTRV2:AtEFR and pTRV2:eGFP were transformed separately into the *A. tumefaciens* EHA105. *Arabidopsis* plants were infected by EHA105 carrying the corresponding constructs using procedures as previously described.^[^ ^49^ ^]^ To investigate RNAi efficiency, RNA was purified from plants collected at 14 dpi. RT‐qPCR was performed to quantify the silencing efficiency using the primer pairs qAtEFRF/qAtEFRR to amplify the AtEFR and UBIF/UBIR to amplify the ubiquitin carboxyl‐terminal hydrolase 22 gene (UBI, AT5G10790).

### Preparation of NemaWater and Mass Spectrum Analysis

Approximate 2 × 10^6^ pre‐J2s were stimulated for 4 h by a hydroponic tomato root culture solution. Then the nematodes were washed three times by ddH_2_O and treated for 4 h with 0.4% resorcinol (Sigma‐Aldrich). NemaWater was centrifuged and then filtered through a 0.22‐µm filter to remove nematodes. The remaining nematodes were collected and washed twice by ddH_2_O, and then used for the extraction of whole nematode protein.^[^ ^28^ ^]^ The NemaWater and whole nematode protein samples were supplemented with 10% SDS to reach a final concentration of 1% SDS. The mixtures were incubated at 95 °C for 5 min and then centrifuged. Proteins in the supernatant were precipitated with Trichloroacetic acid, and the precipitates were resuspended in re‐dissolving solution (8 M Urea, 100 mm Tris‐HCl, pH 8.5). Protein concentrations were determined using the BCA method. Reduction and alkylation of proteins were conducted with TCEP and CAA at 37 °C for 1 h. The Urea was then diluted to 2 M with 100 mm Tris‐HCl. Trypsin was added at a ratio of 1:50 (enzyme: protein, w/w) for overnight digestion at 37 °C. The following day TFA was used to adjust the pH to 6.0 to end the digestion. The supernatants were subjected to peptide purification using a desalting column. All samples were analyzed on an UltiMate3000RSLC nano system coupled online with Q Exactive HF mass spectrometer through a Nanospray Flex ion source (Thermo). MS raw data were analyzed with MaxQuant (1.6.6.0) using the Andromeda database search algorithm. Spectra files were searched against the *M. incognita* protein sequence database downloaded from Uniprot (20231020). Search results were filtered with 1% FDR at both peptide and protein levels. The ratio of relative abundance‐NemaWater to relative abundance‐total proteins of nematodes was calculated for each protein to evaluate whether the protein was enriched in the NemaWater.^[^ ^28^ ^]^

### Plant Immune Response Assay

MAPK activation was determined as described previously.^[^ ^50^ ^]^ Briefly, leaf discs from 30‐day‐old *A. thaliana*, *N. benthamiana*, *O. sativa* and 60‐day‐old *B. napus* were collected and incubated in H_2_O for 4 h. Following this, the leaf discs were treated with various concentrations of cytoplasmic and mitochondrial extracts (5 µg mL^−1^ or 50 µg mL^−1^), NemaWater, MjEF‐Tu (1–30 µg mL^−1^) or 10 µg mL^−1^ truncated derivatives of MjEF‐Tu or vector, 1 µm elf18 or 1 µm flg22 or 1 µm CK peptide (control composed with random amino acids) or H_2_O (Table S1, Supporting Information). After 10 min, total proteins were isolated from six leaf discs using GTEN buffer.^[^ ^43^ ^]^ The sample proteins were denatured, separated, and transferred to a nitrocellulose membrane. After blocking with 5% (w/v) Bovine Serum Albumin (BSA)for 1 h at room temperature, the membranes were incubated with a Phospho‐p44/42 MAPK (erk1/2) (Thr202/Tyr204) Antibody (1:3000 dilution, Cell Signaling Technology, Inc, U.S.A.) (Table 1) in blocking solution for 12 h at 4 °C. Further, the membranes were incubated with an anti‐rabbit horseradish peroxidase‐conjugated secondary anti‐body (Cell Signaling Technology, Inc, U.S.A.) (Table 1). The bands were visualized using the Immobilon Western Chemiluminescent system (Merck) or ChemiDoc Touch Imaging System (Bio‐Rad). The defense‐related gene expression was examined as previously described.^[^ ^20^ ^]^ Briefly, leaf discs from 30‐day‐old Arabidopsis were treated with 10 µg mL^−1^ MjEF‐Tu or 10 µg mL^−1^ vector, 1 µm Nelf18 or 1 µm CK peptide or H_2_O. After 1 h, total RNA was isolated. The expression of four representative defence‐related genes, *FRK1*, *WRKY29*, *WRKY33*, and *CYP81F2*, was determined using RT‐qPCR.^[^ ^22^ ^]^ *A. thaliana ubiquitin carboxyl‐terminal hydrolase 22* gene (UBI, AT5G10790) was used as an internal control and the relative fold change was relative to the expression of H_2_O treated plants. Three independent experiments were performed. The ROS production after treatment with PAMPs was determined as previously described.^[^ ^19^ ^]^ Briefly, leaf discs from 30‐day‐old Arabidopsis were collected and dispatched on a 96‐well plate and incubated in H_2_O for 4 h. Then, the H_2_O was discarded and 100 µL luminol‐based reaction buffer (17 mm luminol, SKU No. 123072‐2.5G, Sigma), 1 mm horseradish peroxidase (SKU No. P8415‐1KU, Sigma) and 10 µg mL^−1^ MjEF‐Tu or 10 µg mL^−1^ vector or 1 µm Nelf18 or 1 µm CK peptide or 1 µm elf18 or 1 µm flg22 or H_2_O were added into each well. Luminescence was measured using a Photek camera system (Thermo) between 0 and 40 min. Callose deposition analysis was performed based on a previous report.^[^ ^22^ ^]^ Briefly, leaf discs from 30‐day‐old Arabidopsis were treated with 1 µM Nelf18 or 1 µm elf18, 1 µm CK peptide or 10 µg mL^−1^ MjEF‐Tu or 10 µg mL^−1^ vector for 24 h. The leaf discs were collected and stained with 0.01% aniline blue in phosphate buffer (pH 7.5). After 1 h, the leaf discs were observed under UV light using an epifluorescence microscope. Callose deposition was quantified from the digital photographs with ImageJ.^[^ ^51^ ^]^

### Infection Assay

For the nematode infection assays, 30‐day‐old Arabidopsis plants were root‐drenched with 2 mL of 1 µm Nelf18, CK peptide or water. After 24 h, 200 pre‐J2s of *M. javanica* were inoculated on the roots of each seedling. Subsequently, the roots were collected, washed, and stained with acid fuchsin at 30 days after inoculation, and the number of females and galls was counted under a microscope. For bacterial growth assays, 30‐day‐old Arabidopsis plants pretreated with 1 µM Nelf18 or CK peptide were infiltrated under vacuum with a suspension of *Pst* DC3000 (concentration of bacterial cells at OD_600_ 0.02 in H_2_O). Bacteria were counted at 4 days post‐inoculation as previously described.^[^ ^11^ ^]^ For CMV infection assays, virus was prepared by grinding *N. benthamiana* leaves, which were infected with the virus by coagroinfiltration with *A. tumefaciens* GV3101 carrying the CMV1, CMV2 and CMV3 constructs.^[^ ^52^ ^]^ 20‐day‐old Arabidopsis plants pretreated with 1 µM Nelf18 or CK peptide were inoculated with CMV by rubbing the inoculum onto a fully expanded mature leaf. 30 days after inoculation, CMV *CP* RNA quantification was performed by RT‐qPCR using the primer pair CMV‐CP‐qF/CMV‐CP‐qR. The *UBI* gene was used as an internal control and the relative fold change was relative to the expression of CK peptide treated plants.

### Statistical Analysis

All values were presented as means ± standard deviation. Prism 9 (GraphPad, USA) and SPSS Statistics V24 (IBM, USA) software were used to analyze the data. Student's *t*‐test was used to analyze differences between two groups. One‐way ANOVA was used to compare three or more groups. Probability values less than 0.05 was considered statistically significant.

## Conflict of Interest

The authors declare no conflict of interest.

## Author Contributions

B.R.L. and S.Z.H. contributed equally to this work and should be considered co‐first authors. K.Z. and B.L. contributed to concept and design; B.L., S.H., Z.L., Q.H., H.S., and T.F. contributed to method and investigate; K.Z., B.L., and J.L. contributed to the analysis, and interpretation of data; B.L., S.H., G.G., and K.Z. contributed to write, review, and/or revision of the manuscript.

## Supporting information



Supporting Information

## Data Availability

The data that support the findings of this study are available from the corresponding author upon reasonable request.
