# Supplementary material for: Mitochondrial Protein MjEF‐Tu is Secreted into Host Plants by Nematodes Eliciting Immune Signaling and Resistance
Source: Adv Sci (Weinh). 2025 Jan 30;12(11):2412968. doi: 10.1002/advs.202412968 (PMC11923865; doi:10.1002/advs.202412968)
Supplement: Supplementary file 1 — Supporting Information [file ADVS-12-2412968-s001.docx]

Supporting information


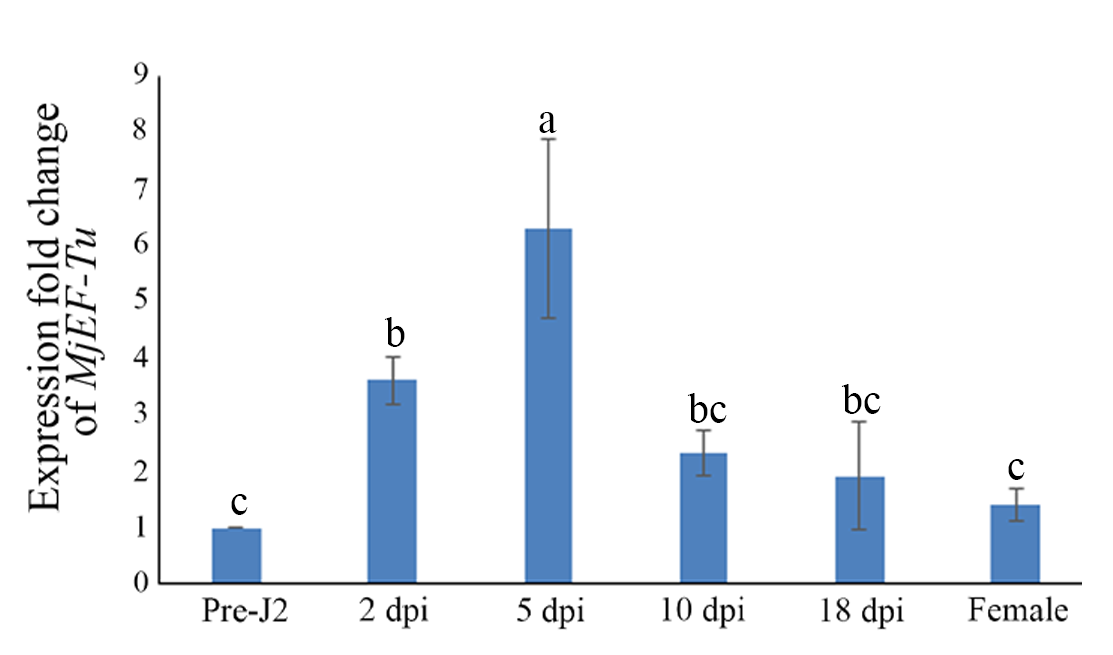


**Figure S1. The developmental expression pattern of MjEF-Tu via quantitative real-time polymerase chain reaction analysis at six different life stages of Meloidogyne javanica**.

The fold change values were calculated using the 2^-ΔΔCT^ method and presented as the change in mRNA level at various nematode developmental stages relative to that of the pre-parasitic second stage juveniles (pre-J2). dpi, days post-inoculation.


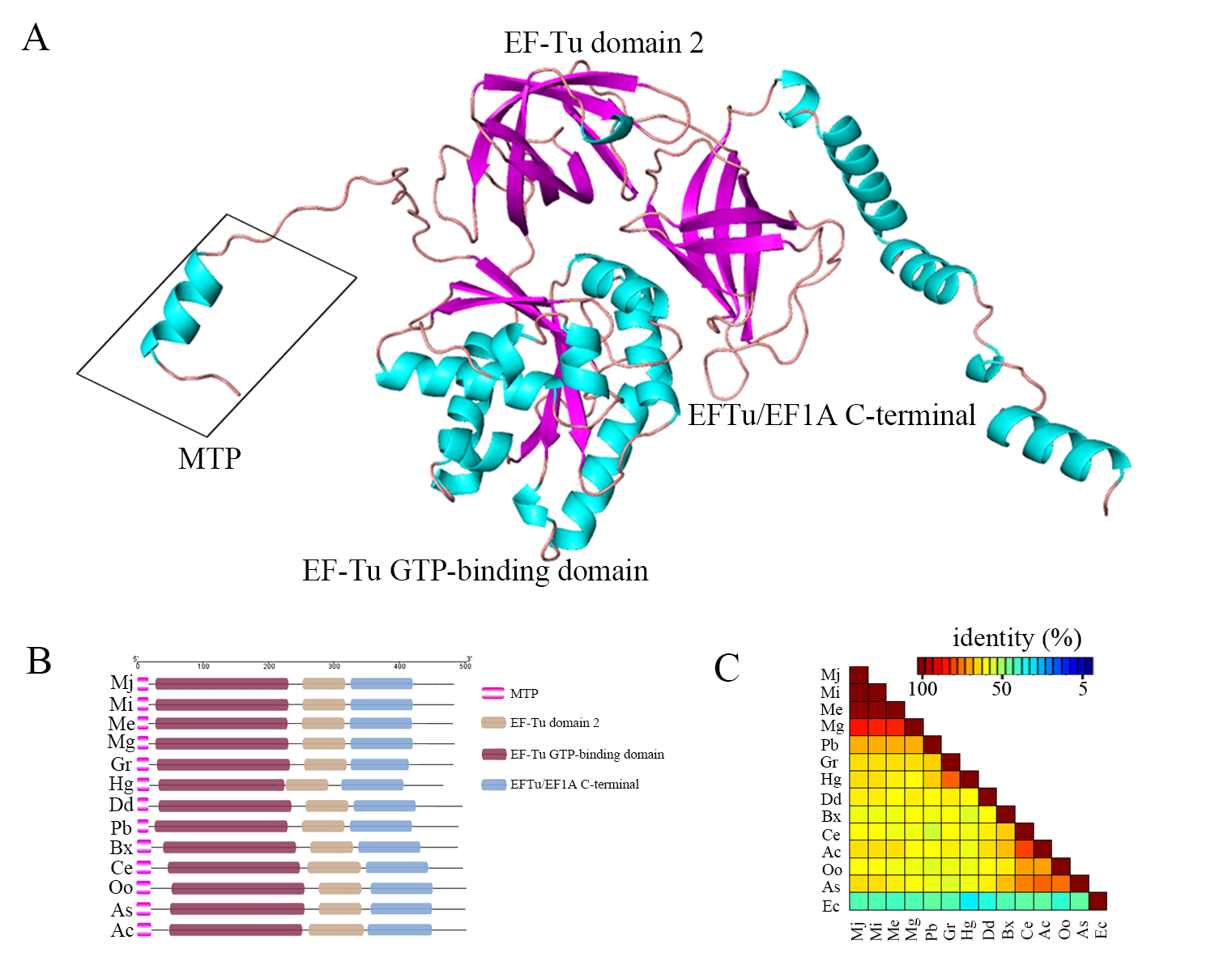


**Figure S2. Sequence analyses of the *M* Meloidogyne *javanica* gene *MjEF-Tu***.

A) Structure of MjEF-Tu predicted from amino acid sequence with trRosetta. MTP, mitochondrial transit peptide. B) The domain diagram of EF-Tu proteins. Different colours represent different domains; C) Sequence identity matrix for EF-Tu proteins; Ec, *Escherichia coli*; Mj, *Meloidogyne javanica*; Mi, *M. incognita*; Me, *M. enterolobii*; Mg, *M. graminicola*; Gr, *Globodera rostochiensis*; Hg, *Heterodera glycines*; Dd, *Ditylenchus destructor*; Pb, *Pratylenchus brachyurus*; Bx, *Bursaphelenchus xylophilus*; Ce, *Caenorhabditis elegans*; As, *Ascaris suum*; Ac, *Ancylostoma ceylanicum*; Oo, *Onchocerca ochengi.* Mitochondrial transit peptides is highlighted in red box.


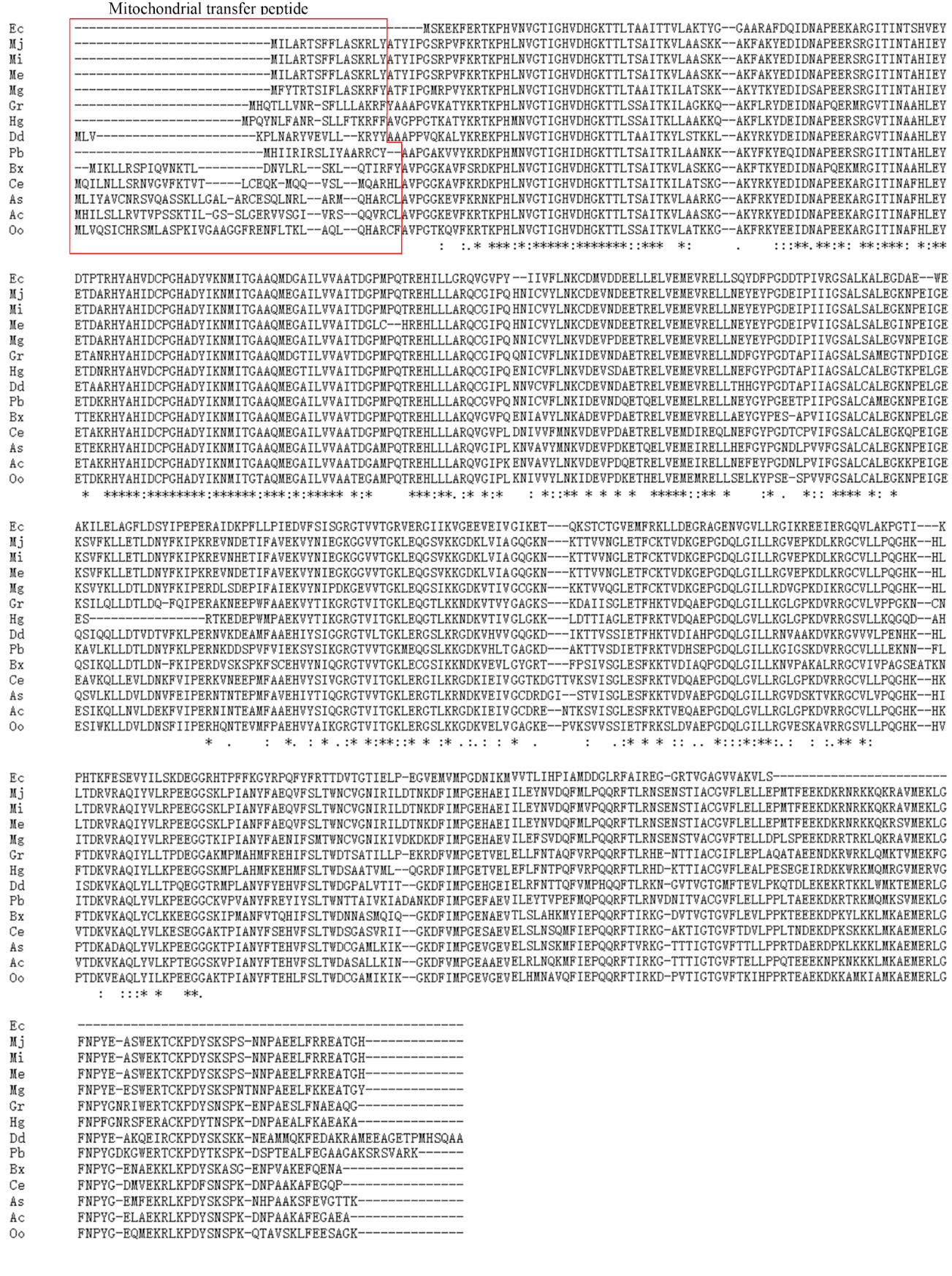


**Figure S3. Multiple sequence alignment of EF-Tu from different nematodes.**

Ec, *Escherichia coli*; Mj, *Meloidogyne javanica*; Mi, *M. incognita*; Me, *M. enterolobii*; Mg, *M. graminicola*; Gr, *Globodera rostochiensis*; Hg, *Heterodera glycines*; Dd, *Ditylenchus destructor*; Pb, *Pratylenchus brachyurus*; Bx, *Bursaphelenchus xylophilus*; Ce, *Caenorhabditis elegans*; As, *Ascaris suum*; Ac, *Ancylostoma ceylanicum*; Oo, *Onchocerca ochengi.* Mitochondrial transit peptides is highlighted in red boxes.


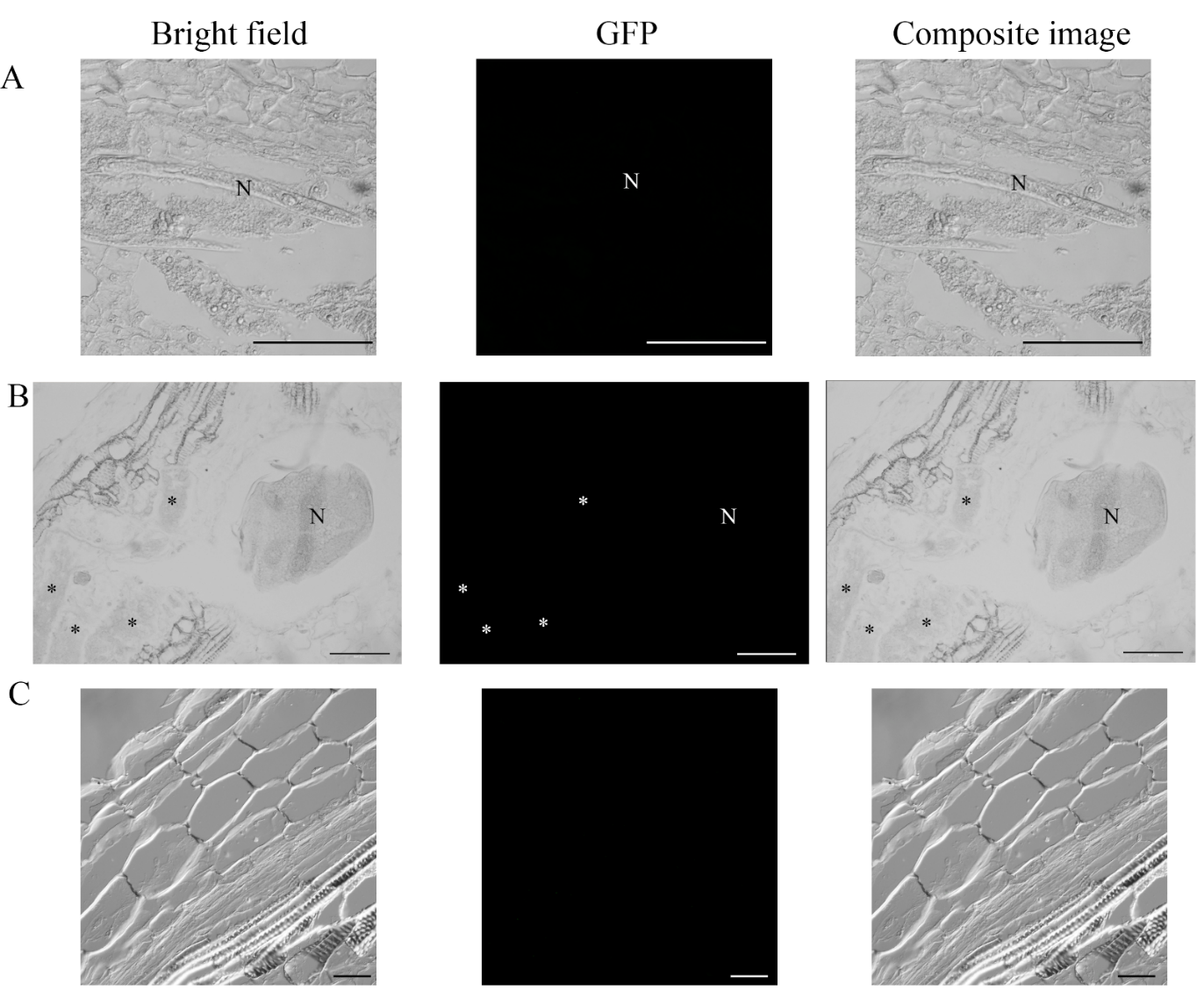


**Figure S4.** **Immunodetection of the MjEF-Tu protein in sectioned tomato galls or healthy roots**.

A-B) Galls containing a nematode at 5 days and 18 days post-inoculation (dpi) that were incubated with only Alexa Fluor 488-conjugated secondary antibody show no signals; C) Healthy tomato roots incubated with anti-MjEF-Tu antibody and Alexa Fluor 488-conjugated secondary antibody also show no signals; N, nematode. *, giant cell. Bar=100 μm.


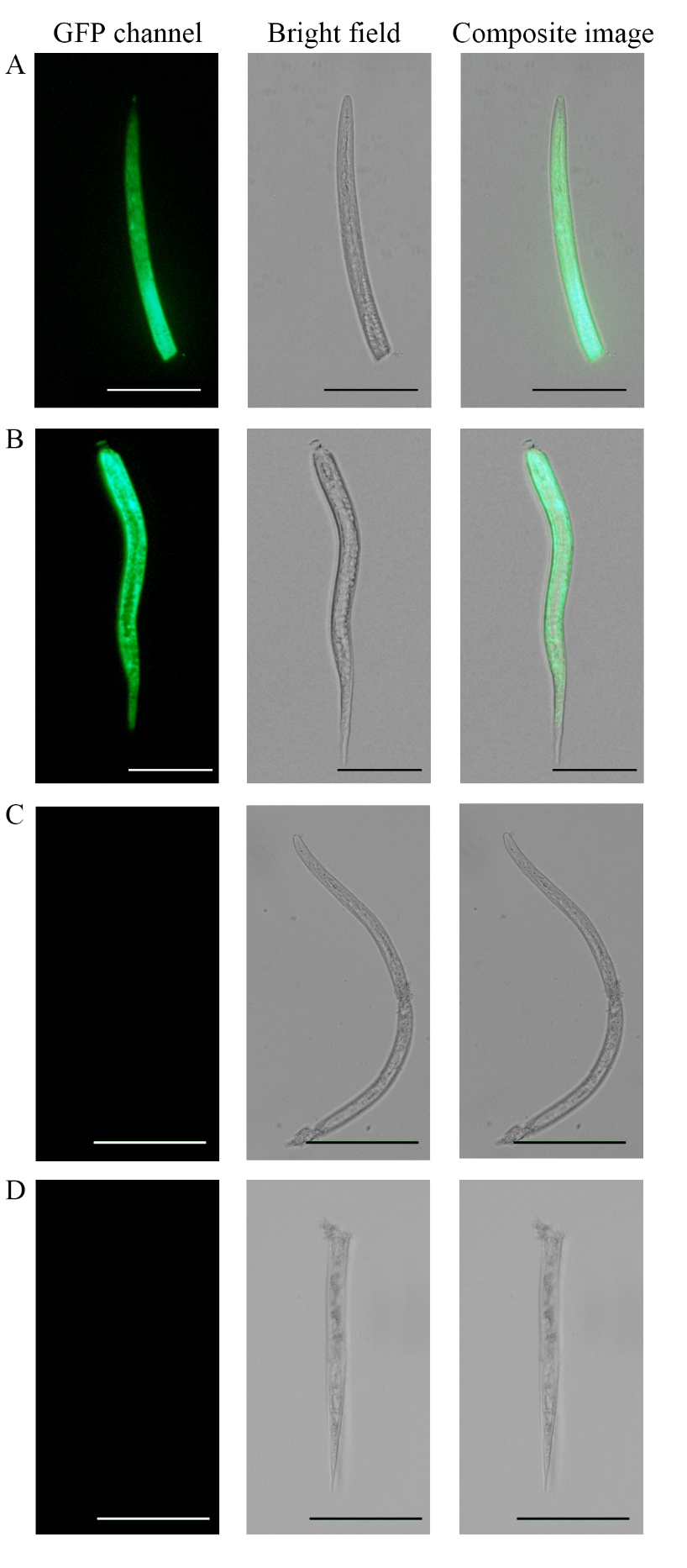


**Figure S5. Immunolocalization of MjEF-Tu in nematodes.**

A-B) Pre-parasitic second-stage juvenile (pre-J2) showing the protein ubiquitously expressed throughout the nematode and C-D) absence of signal in pre-J2 only by Alexa Fluor 488-conjugated secondary antibody. Bars = 50 μm.


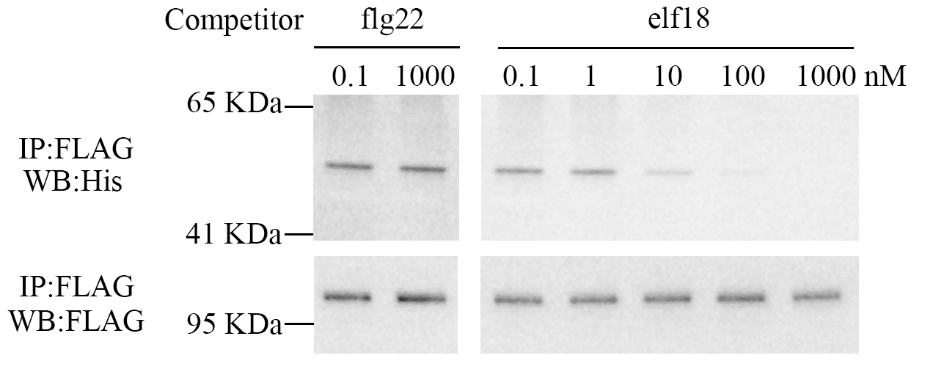


**Figure S6. Interaction between AtEFR:Flag and MjEF-Tu:His in the presence of different concentrations of unlabeled elf18.**

Immunodetected MjEF-Tu:His bands were quantified using the ImageJ software. Equal loading was checked by immunodetection of AtEFR:Flag.


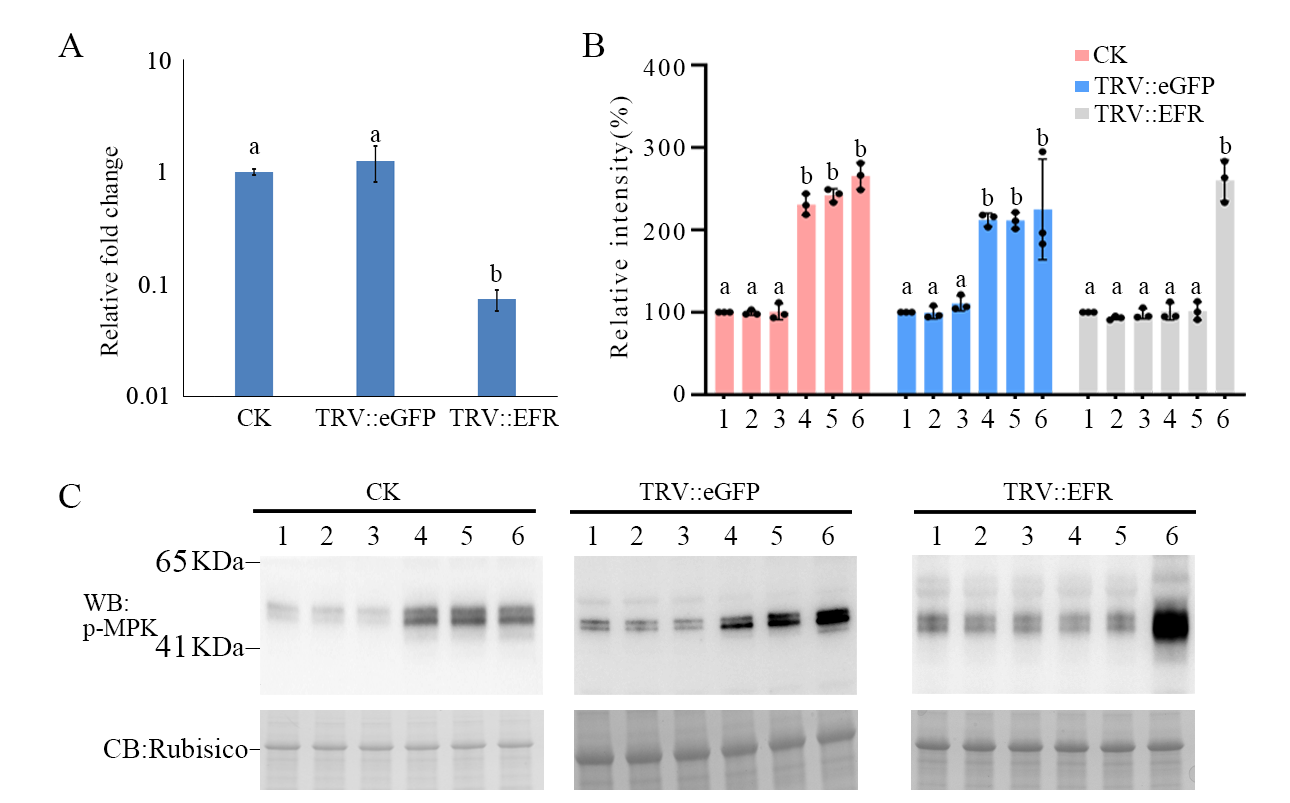


**Figure S7.** **TRV-mediated *AtEFR* silencing decreases plant sensitivity to MjEF-Tu.**

A) RT-qPCR assays of expression levels of *AtEFR* in *Arabidopsis* plants collected from noninfiltrated plants (CK), pTRV2:eGFP(TRV::eGFP) and pTRV2:AtEFR(TRV::EFR) agroinfiltrated plants; B-C) The activation of MAPKs in noninfiltrated plants, pTRV2:eGFP and pTRV2:AtEFR agroinfiltrated plants after treatment with H_2_O (Lane 1), protein purified from *Escherichia coli* carrying pET28a vector (Lane 2), eGFP protein (Lane 3), MjEF-Tu (Lane 4), elf18 (Lane 5) and flg22 (Lane 6), respectively. CB, coomassie brilliant blue staining of total proteins (loading control). Data are presented as means±SD, and the mean values marked with different letters are significantly different from each other as determined by Duncan’s multiple range test (P < 0.05).


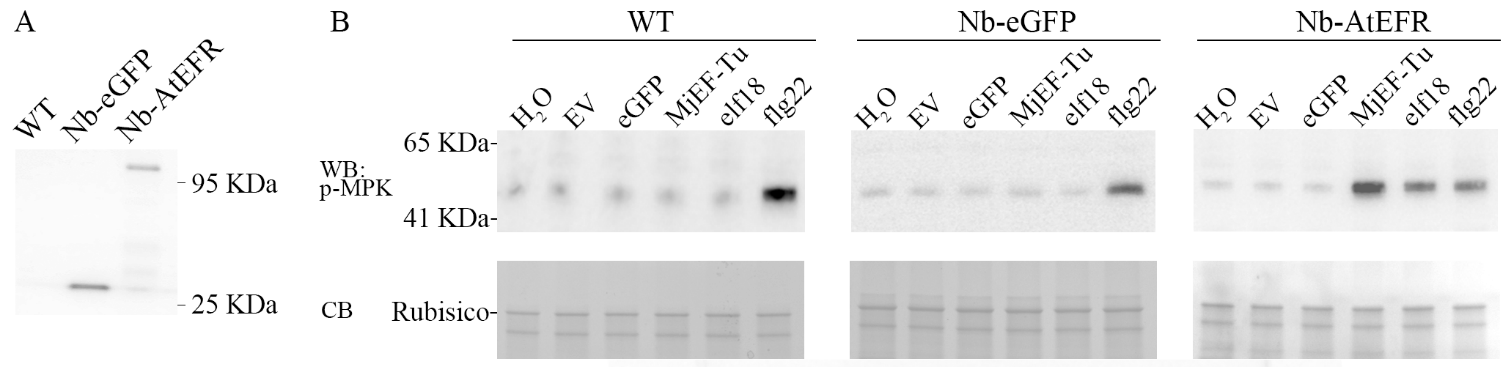


**Figure S8. Activation of MAPKs after treatment with MjEF-Tu in *Nicotiana benthamiana* transiently expressing *AtEFR.***

A) Western blot analysis of proteins from *N. benthamiana* transiently expressing *eGFP* (Nb-eGFP) and *AtEFR* (Nb-AtEFR), showed a ~28 KDa and 110 KDa bands; B) Immunoblot analysis of phosphorylated MAPKs (p-MPK) in wild-type *N. benthamiana* (WT), Nb-GFP and Nb-AtEFR. H_2_O, EV, eGFP, MjEF-Tu, elf18 and flg22 represent treatment with H_2_O, protein purified from *Escherichia coli* carrying pET28a vector, eGFP protein, MjEF-Tu protein, elf18 and flg22 peptide. CB, coomassie brilliant blue staining of total proteins (loading control).


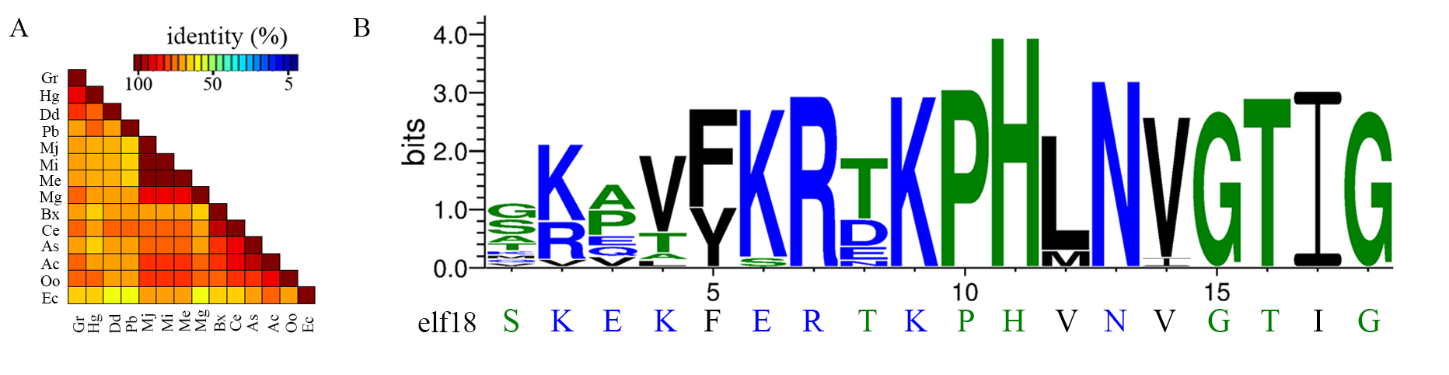


**Figure S9. Sequence analyses of a conserved 18-amino acid sequence within N terminus of EF-Tu proteins (Nelf18) from different nematodes.**

A) Sequence identity matrix for Nelf18 from different nematodes; Ec, *Escherichia coli*; Mj, *Meloidogyne javanica*; Mi, *M. incognita*; Me, *M. enterolobii*; Mg, *M. graminicola*; Gr, *Globodera rostochiensis*; Hg, *Heterodera glycines*; Dd, *Ditylenchus destructor*; Pb, *Pratylenchus brachyurus*; Bx, *Bursaphelenchus xylophilus*; Ce, *Caenorhabditis elegans*; As, *Ascaris suum*; Ac, *Ancylostoma ceylanicum*; Oo, *Onchocerca ochengi*; B) The Nelf18 logo from different nematode species. Blue, green and black characters represent hydrophilic, neutral and hydrophobic amino acids.


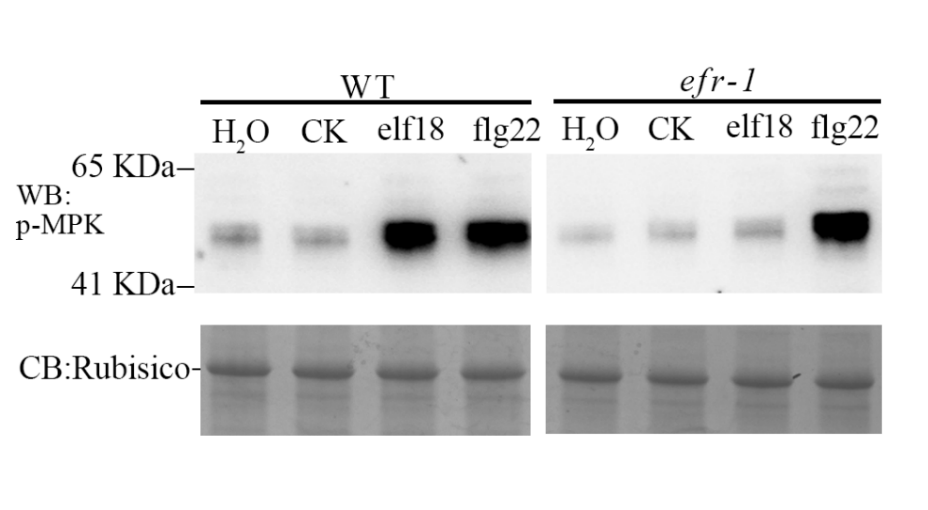


**Figure S10. Immunoblot analysis of phosphorylated MAPKs (p-MPK) in WT and *efr-1* *Arabidopsis thaliana* after treatment with different peptides.**

H_2_O, CK, elf18 and flg22 represent treatment with H_2_O, CK peptide that is composed with random amino acids (SAHHAEHHHPHVHHAHHA), elf18 and flg22 peptide, respectively; WT, wild-type Arabidopsis; *efr-1*, Arabidopsis lacking the EF-Tu receptor (EFR).





**Figure S11. Purification of pET28a (EV), recombinant MjEF-Tu, truncated derivatives of MjEF-Tu and eGFP.** Sodium dodecyl sulfate-polyacrylamide gel electrophoresis analysis stained with Coomassie brilliant blue; M: the protein’s standard molecular weight.

**Table S1. List of peptides used in the study.**

| Peptide | Organism | Peptide sequence |
| --- | --- | --- |
| Elf18 | *Escherichia coli* | Ac-SKEKFERTKPHVNVGTIG |
| NElf18^Mj^ | *Meloidogyne javanica* | Ac-SRPVFKRTKPHLNVGTIG |
| NElf18^Mg^ | *M. graminicola* | Ac-MRPVYKRTKPHLNVGTIG |
| NElf18^pr^ | *Pratylenchus brachyurus* | Ac-AKVVYKRDKPHMNVGTIG |
| NElf18^Hg^ | *Heterodera glycines* | Ac-TKATYKRTKPHMNVGTIG |
| Flg22 | *E. coli* | QRLSTGSRINSAKDDAAGLQIA |
| CK | CK | SAHHAEHHHPHVHHAHHA |

**Table S2. List of primers used in the study.**

| Primer | Sequence (5’-3’) | Reference |
| --- | --- | --- |
| **FRK1F** | **TGCAGCGCAAGGACTAGAG** | Jaouannet et al.^1^ |
| **FRK1R** | **ATCTTCGCTTGGAGCTTCTC** |  |
| **WRKY3F** | **GCTGCTATTGCTGGTCACTCC** |  |
| **WRKY3R** | **GGTCTCCTCGTTTGGTTCTTCC** |  |
| **WRKY2F** | **ATCCAACGGATCAAGAGCTG** |  |
| **WRKY2R** | **GCGTCCGACAACAGATTCTC** |  |
| **CYP8F** | **GTGAAAGCACTAGGCGAAGC** |  |
| **CYP8R** | **ATCCGTTCCAGCTAGCATCA** |  |
| **UBIF** | **GCCAAAGCTGTGGAGAAAAG** |  |
| **UBIR** | **TGTTTAGGCGGAACGGATAC** |  |
| **PAD4F** | **GCCGCTTTCACCGCACTTTGG** |  |
| **PAD4R** | GAGAGATTGGTTTCCGAGCAGAGG |  |
| **MjEF-TuF** | TACGCTCATATGATTTTGGCAAGGACTTCTTT | This study |
| **MjEF-TuR** | ACGGGATCCTCAATGTCCAGTTGCTTCCCTTC |  |
| qMjEFTuF | TACCACAGCAGCGCTTTACTC |  |
| qMjEFTuR | GGCTTGCAAGTCTTTTCCCAA |  |
| isElfF | ATGGAAGTTAGAGAACTTTTGAATG |  |
| isElfR | AGTGACAACTCCACCTTTTCC |  |
| RNAiElfF | ggatcctaatacgactcactatagggATGGAAGTTAGAGAACTTTTGAATG |  |
| RNAiElfR | ggatcctaatacgactcactatagggAGTGACAACTCCACCTTTTCC |  |
| p28elfF | ggacagcaaatgggtcgcATGATTTTGGCAAGGACTTCTTT | This study |
| p28elfR | tggtggtggtggtggtgATGTCCAGTTGCTTCCCTTCTAA |  |
| p28elfF-4 | ggacagcaaatgggtcgcGCGACCTACATTCCTGGTTCA |  |
| p28elfF-3 | ggacagcaaatgggtcgcCCAAAAGATCTTAAAAGGGGATGT |  |
| p28elfF-6 | ggacagcaaatgggtcgcTACATTCCTGGTTCAAGGCCAGTTTTC |  |
| p28elfR-6 | tggtggtggtggtggtgAGATAATGCAGAGCCAATAATTATC |  |
| p28gfpF | ggacagcaaatgggtcgcATGGTGAGCAAGGGCGAGGAG |  |
| p28gfpR | tggtggtggtggtggtgCTTGTACAGCTCGTCCATGCC |  |
| p28elfF-7 | ggacagcaaatgggtcgcAAACGTGAGGTTAATGATGAAACT |  |
| p28elfF-8 | ggacagcaaatgggtcgcAAAAACATGATCACCGGAGCTG |  |
| p28elfR-8 | tggtggtggtggtggtgACGTTTAGGAATTTTAAAATAATTA |  |
| p28gfpF | ggacagcaaatgggtcgcATGGTGAGCAAGGGCGAGGAG |  |
| p28gfpR | tggtggtggtggtggtgCTTGTACAGCTCGTCCATGCC |  |
| BKGFPF | caagcctcctgaaagatgGAATTCCCGGGATCCCATATGGTGAGCAAGGGCGAGGAG |  |
| BKGFPR | ctagttatgcggccgCTTGTACAGCTCGTCCATGCC |  |
| ADRFPF | agctttgcaaagatgGAATTCCCGGTCGACATGGTGAGCAAGGGCGAGGAG |  |
| ADRFPR1 | gagctcgatggatccTCAGGCGCCGGTGGAGTGGCGG |  |
| BKELFF | agcctcctgaaagatgATGATTTTGGCAAGGACTTC |  |
| BKELFR | gcccttgctcaccatATGTCCAGTTGCTTCCCTTC |  |
| BKELF-mpF | agcctcctgaaagatgGCGACCTACATTCCTGGTTCA |  |
| BKMPF | agcctcctgaaagatgATGATTTTGGCAAGGACTTCTTTTTTTCTAGCTTCAAAGCGCTTATATGCGatggtgagcaagggc |  |
| BKMPR | gcccttgctcaccatCGCATATAAGCGCTTTGAAGCTAGAAAAAAAGAAGTCCTTGCCAAAATCATcatctttcaggaggct |  |
| ARScF | aagctttgcaaagatggaaATGCTTTCACTACGTCAATCTATAAG |  |
| ARScR | tcgcccttgctcaccatgtcGTGATGGTGGTCATCATTTGGAA |  |
| TRV-efrF | agtaaggttaccgaattcCAATGCTCTCACGTTGCTTCTT | This study |
| TRV-efrR | ctcgagacgcgtgagctcTCATGTTCAAGTACTGAAGCC |  |
| qAtEFRF | GGCTATGCCGCGCCAGAGTAT |  |
| qAtEFRR | AACCCCTCATCAATGGCGTTGC |  |
| CMV-CP-qF | GTTCCTGCCTCCTCGGACTTATC |  |
| CMV-CP-qR | GGAATGCGTTGGTGCTCGATGTC |  |
| qMjActinF | TGTCAATGTCGCACTTCATGATC | Lin et al.^2^ |
| qMjActinR | CCGTTGCCCAGAATCTCTCTT |  |

**Supporting Methods and Results**

**Methods**

**Isolation of fungi and bacteria from *Meloidogyne javanica***

Approximately 5000 pre-parasitic second-stage juveniles (pre-J2s) were collected. A total of 200 μL sterile water was added into the tube containing nematodes, followed by shaking on a shaker for 10 min. Subsequently, the mixture was centrifuged at 14000 g for 5 min. Then, the 10-μL supernatant was diluted to 1 mL. A 100 μL aliquot of the dilution was spread onto LB agar plates for bacteria and PDA agar plates for fungi, respectively. The plates were then incubated at 25℃ for 3 days. Colonies were transferred from the plates to the liquid LB and PDA medium. For bacterial identification, 16S rDNA region was amplified using the primer pair 27F/1492R, while for fungal identification, the internal transcribed spacer of ribosomal RNA gene was amplified by the primer pair ITS1F/ITS4R. The PCR products were cloned into pMD-19T vector and sequenced. Total proteins were isolated using GTEN buffer.

**Results**

More than 200 colonies grew in LB agar plates, out of which 14 colonies were randomly selected for PCR and sequencing. In PDA agar plates, only 4 colonies grew, and all of them were used for PCR and sequencing.

According to the sequencing results, among 14 colonies from LB plates, 11 colonies belonged to *Ralstonia* sp., 2 colonies belonged to *Dickeya* sp. and 1 colony belonged to *Kitasatospora* sp. And, all 4 colonies from PDA belonged to *Saccharomycetales* sp.

Consequently, proteins from *Ralstonia* sp. and *Saccharomycetales* sp. were used to determine the specificity of the anti-MjEF-Tu antibody.

**Reference**

[1] M. Jaouannet, M. Magliano, M.J. Arguel, M. Gourgues, E. Evangelisti, P. Abad, and M.N. Rosso. *Mol.* *Plant-Microbe Interact.* **2013**, *26*, 97-105.

[2] B.R. Lin, K. Zhuo, S.Y. Chen, L.L. Hu, L.H. Sun, X.H. Wang, L.H. Zhang and J.L. Liao. *New Phytol.* **2016**, *209*, 1159-1173.
